# KNeMAP: a network mapping approach for knowledge-driven comparison of transcriptomic profiles

**DOI:** 10.1093/bioinformatics/btad341

**Published:** 2023-05-24

**Authors:** Alisa Pavel, Giusy del Giudice, Michele Fratello, Leo Ghemtio, Antonio Di Lieto, Jari Yli-Kauhaluoma, Henri Xhaard, Antonio Federico, Angela Serra, Dario Greco

**Affiliations:** Faculty of Medicine and Health Technology, Tampere University, 33520 Tampere, Finland; BioMediTech Institute, Tampere University, 33520 Tampere, Finland; Finnish Hub for Development and Validation of Integrated Approaches (FHAIVE), 33520 Tampere, Finland; Faculty of Medicine and Health Technology, Tampere University, 33520 Tampere, Finland; BioMediTech Institute, Tampere University, 33520 Tampere, Finland; Finnish Hub for Development and Validation of Integrated Approaches (FHAIVE), 33520 Tampere, Finland; Faculty of Medicine and Health Technology, Tampere University, 33520 Tampere, Finland; BioMediTech Institute, Tampere University, 33520 Tampere, Finland; Finnish Hub for Development and Validation of Integrated Approaches (FHAIVE), 33520 Tampere, Finland; Drug Research Program, Division of Pharmaceutical Biosciences, Faculty of Pharmacy, University of Helsinki, 00790 Helsinki, Finland; Mental Health Services, Landspitali University Hospital, 101 Reykjavik, Iceland; Drug Research Program, Division of Pharmaceutical Biosciences, Faculty of Pharmacy, University of Helsinki, 00790 Helsinki, Finland; Drug Research Program, Division of Pharmaceutical Biosciences, Faculty of Pharmacy, University of Helsinki, 00790 Helsinki, Finland; Faculty of Medicine and Health Technology, Tampere University, 33520 Tampere, Finland; BioMediTech Institute, Tampere University, 33520 Tampere, Finland; Finnish Hub for Development and Validation of Integrated Approaches (FHAIVE), 33520 Tampere, Finland; Tampere Institute for Advanced Study, 33520 Tampere, Finland; Faculty of Medicine and Health Technology, Tampere University, 33520 Tampere, Finland; BioMediTech Institute, Tampere University, 33520 Tampere, Finland; Finnish Hub for Development and Validation of Integrated Approaches (FHAIVE), 33520 Tampere, Finland; Tampere Institute for Advanced Study, 33520 Tampere, Finland; Faculty of Medicine and Health Technology, Tampere University, 33520 Tampere, Finland; BioMediTech Institute, Tampere University, 33520 Tampere, Finland; Finnish Hub for Development and Validation of Integrated Approaches (FHAIVE), 33520 Tampere, Finland; Institute of Biotechnology, University of Helsinki, 00790 Helsinki, Finland; Division of Pharmaceutical Biosciences, Faculty of Pharmacy, University of Helsinki, 00790 Helsinki, Finland

## Abstract

**Motivation:**

Transcriptomic data can be used to describe the mechanism of action (MOA) of a chemical compound. However, omics data tend to be complex and prone to noise, making the comparison of different datasets challenging. Often, transcriptomic profiles are compared at the level of individual gene expression values, or sets of differentially expressed genes. Such approaches can suffer from underlying technical and biological variance, such as the biological system exposed on or the machine/method used to measure gene expression data, technical errors and further neglect the relationships between the genes. We propose a network mapping approach for knowledge-driven comparison of transcriptomic profiles (KNeMAP), which combines genes into similarity groups based on multiple levels of prior information, hence adding a higher-level view onto the individual gene view. When comparing KNeMAP with fold change (expression) based and deregulated gene set-based methods, KNeMAP was able to group compounds with higher accuracy with respect to prior information as well as is less prone to noise corrupted data.

**Result:**

We applied KNeMAP to analyze the Connectivity Map dataset, where the gene expression changes of three cell lines were analyzed after treatment with 676 drugs as well as the Fortino *et al.* dataset where two cell lines with 31 nanomaterials were analyzed. Although the expression profiles across the biological systems are highly different, KNeMAP was able to identify sets of compounds that induce similar molecular responses when exposed on the same biological system.

**Availability and implementation:**

Relevant data and the KNeMAP function is available at: https://github.com/fhaive/KNeMAP and 10.5281/zenodo.7334711.

## 1 Introduction

A fundamental challenge in compound safety and efficacy assessment is to understand the multi-scale mechanistic effects that compounds have on genes, cells, tissues, and organisms. Toxicogenomics approaches can be used to characterize the mechanism of action (MOA) of a compound (Gao *et al.* 2021), through the use of transcriptomics (Federico *et al.* 2020, Kinaret *et al.* 2020b, Serra *et al.* 2020). In addition, the comparison of molecular alteration profiles allows to identify similarities between phenotypic entities and to make conclusions about possible phenotypic changes of an exposure (Kinaret *et al.* 2020b). Transcriptomics data are complex and prone to technical and biological variability and noise (Raser and O’Shea 2005, Freytag *et al.* 2015, Federico *et al.* 2020, Fratello *et al.* 2022). Therefore many variables need to be considered when comparing expression profiles, especially coming from different datasets or (biological) systems.

Methods to compare gene expression or gene expression alteration profiles aim to analyze lists of genes ordered by their expression levels as measured by DNA microarrays or RNA sequencing (Federico *et al.* 2020, Kinaret *et al.* 2020b). A common metric used for this is the correlation (Freytag *et al.* 2015, Serra *et al.* 2018, Serra *et al.* 2020). Differential analysis or the comparison of deregulated genes is another method, where the affected genes are compared with respect to a control, instead of using the expression values directly (Marwah *et al.* 2019, Federico *et al.* 2020). In this case, the lists of deregulated genes are directly compared to highlight differences and commonalities. Alternatively their functional profiles are compared through pathway enrichment (Federico *et al.* 2020, Serra *et al.* 2022b).

The approach suggested in this study, a network mapping approach for knowledge-driven comparison of transcriptomic profiles (KNeMAP), builds on the assumption that genes can be grouped together based on higher level classifications, such as functions, processes or evolutionary origin. Therefore the individual gene view is replaced by a “similar gene” view, where instead of considering genes individually, a set of genes are grouped together based on multi-level prior knowledge. This gene grouping is used to create a feature vector for each experimental instance, which can be used in downstream analysis, such as clustering or machine learning (ML) applications, where often a numeric feature vector is needed as input (Serra *et al.* 2020, Fratello *et al.* 2022). This is in contrast to many functional enrichment applications, where individual pathway names are returned, that cannot be directly provided as input to such downstream ML applications.

In addition since KNeMAP is prior knowledge dependent, new feature vectors can be computed for new data, without the need to re-process existing data, since the feature vectors as long as computed from the same prior knowledge are comparable between each other. For the same reason it is also possible to compare exposure fingerprints via KNeMAP across datasets. Another difference to traditional functional enrichment is that we define gene similarity as multi-view, across multiple different data layers, capturing functional, interactional, and associational gene (product) similarities.

Here, we showcase the effectiveness of the KNeMAP method by applying it on the CMap (Lamb *et al.* 2006) dataset to compare the transcriptomic profiles of drugs across three different cell lines (biological systems), as well as the Fortino *et al.* (2022) dataset to compare the transcriptomic profiles of engineered nanomaterials (ENMs) across two different human cell lines. In addition, we compare the CMap (Lamb *et al.* 2006) and Fortino *et al.* data with each other to identify for each ENM, the drug that shows the most similar transcriptomic alterations across all biological systems. We also compare our method with three other approaches based on correlation of the gene expression fold changes (in comparison to the control gene expression), gene deregulation analysis as well as a Gene Set Enrichment Analysis (GSEA)-based methodology (Subramanian *et al.* 2005, Iorio *et al.* 2010).

## 2 Materials and methods

### 2.1 Data collection and prior network

**Figure 1.**
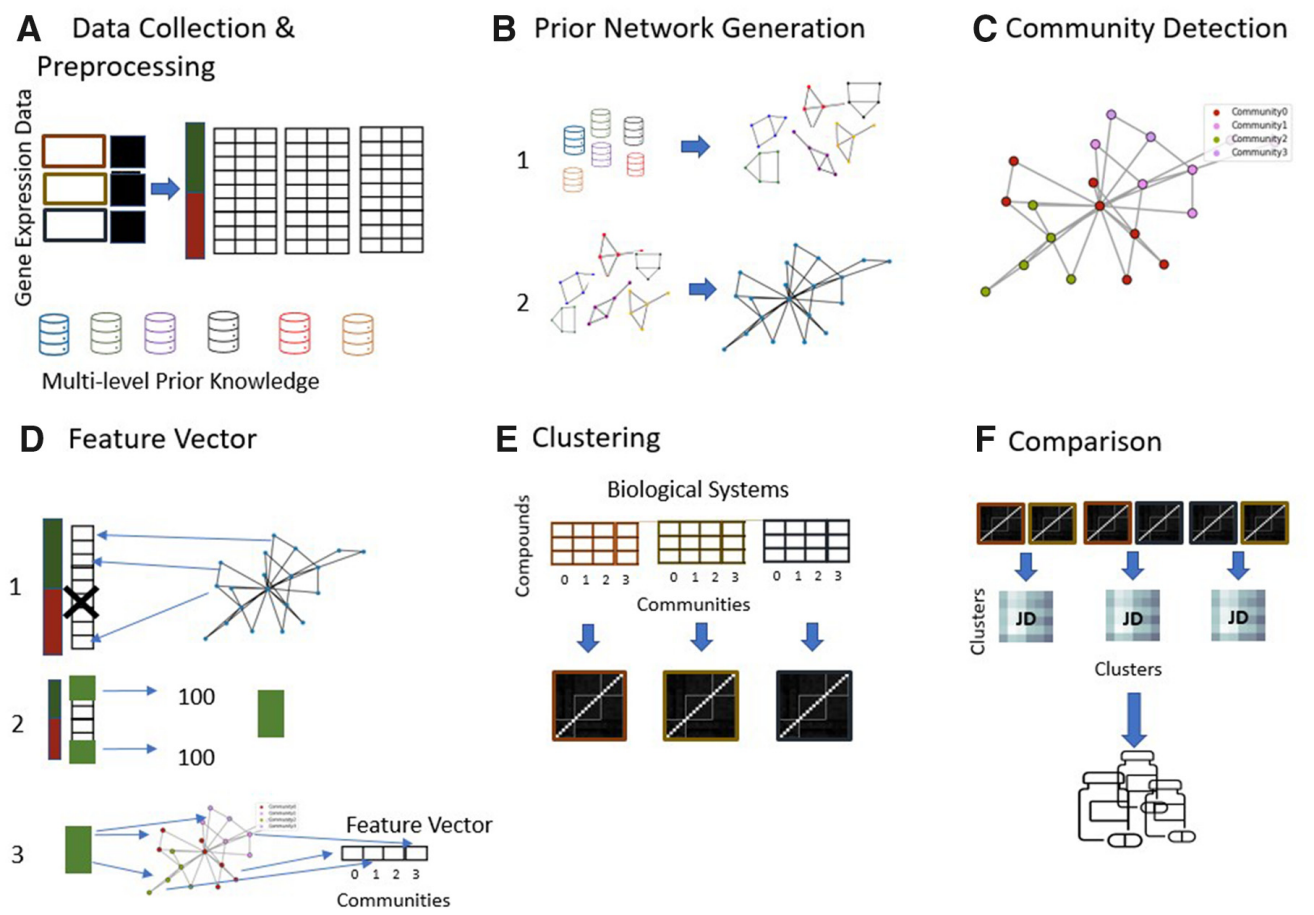
Description of the proposed methodology. (A) The collection and pre-processing of the gene expression data. The values are sorted by their ±*logFC* * −*log*(*Pval*) (FCP) values. In addition, different layers of gene (product) information are collected, such as protein family, homolog, protein-protein interaction (PPI) information as well as associations to phenotypes, compounds, and gene ontology (GO) (The Gene Ontology Consortium 2021) terms. (B) The individual gene (product) information data types are converted into gene–gene similarity networks (1). The individual networks are merged into a single weighted gene–gene similarity network, the prior network (2). (C) The prior network is partitioned into communities. (D) For each exposure a feature vector is created. The gene expression data are filtered to only include genes contained in the prior network (1). The genes are sorted by their up/down regulation and the top (up-regulated) and bottom (down-regulated) 100 genes are selected (2). These 200 genes are mapped onto the prior network partitions (communities). For each exposure a feature vector is created, whose length is equal to the number of detected communities and its values indicate the fraction of the most affected 200 genes falling into each community (3). (E) The feature vectors are used to cluster the compounds for each biological system. (F) The clusters are compared between the biological systems, via a jaccard index.

In order to investigate the difference between the transcriptomic alterations induced by small molecules on different biological systems, we downloaded microarray data including a set of compounds, tested on different systems (Fig. 1A), as described in Lamb *et al.* (2006) (CMap) and as described in Fortino *et al.* (2022). The processing of the data is described in the Supplementary Materials (Methods—Collection of Expression Data and Pre-Processing).

#### 2.1.1 Prior network creation and community detection

In order to build a robust gene network, we collected multiple data layers and datasets, covering different aspects of a gene's function, relationships, and structure (Fig. 1A). By combining these data, we created a weighted network that captures multiple views of “gene similarity.” For example two genes can be considered as similar, based on their structural or ancestral similarities, on their functional similarities (e.g. takes part in the same pathway) or on a higher level, such as that genes are associated with the same or closely related phenotypes. A similar approach is applied in multi-omics, where data from different omics technologies are combined in order to generate a more complete view of the analyzed data (Serra *et al.* 2015, Rappoport and Shamir 2018, Mitra *et al.* 2020). The data used to create the prior network is described in the Supplementary Materials (Methods—Prior Network Data Collection). Which has been integrated into a Knowledge Graph framework (Pavel *et al.* 2022), the Unified Knowledge Space (UKS), which has been previously described in Pavel *et al.* (2021a,b) and Federico *et al.* (2022).

##### 2.1.1.1 Prior network

For each of the data types collected (Paralog, Homolog, Protein Family, Protein Sub-Family, Chemical associations, Disease associations, Pathways, Biological Process, Molecular Function, Cellular Component, PPI) a single gene–gene similarity network was created (Fig. 1B1). For data representing gene–gene edges in the UKS, such as contained in the protein–protein interaction layer a gene–gene similarity network was constructed by retrieving the interactions and assigning as weights the number of data sources supporting this edge. This approach of unifying gene networks has already proven to be effective, as described in Pavel *et al.* (2021a,b). The other type of data, representing gene–entity edges, such as gene–disease associations or gene–pathway associations were converted into a gene–gene similarity network. Here an edge represents two genes that are associated with the same entity (e.g. a disease) and the edge weight represents how many shared entities the pair of genes has, similarly to the approach described in Federico *et al.* (2022). After the individual gene similarity networks were created, their edge weights were scaled to be in (0,1), where a value close to 1 represents a strong similarity and a value close to 0 represents a weak similarity. This was performed in order to merge the individual networks into a combined gene similarity network. The individual networks were merged in a hierarchical, data-driven fashion. First the individual networks edge similarity was assessed, based on a combined distance on their binary edges. The aim was to first merge data layers, which span similar areas, therefore it was only considered if an edge is present or not and not their computed edge weights, which are first considered in the merging process. The combined distance was created by summing the jaccard distance matrix, the SMC (Simple Matching Coefficient, also known as Rand similarity) distance matrix and a distance matrix computed from the percentage of shared edges (1-fraction of shared edges) (Pavel *et al.* 2021a,b). All three distance matrices were weighted equally and the resulting distance matrix was scaled to be in (0,1). On this combined matrix, hierarchical clustering was performed with *scipy.cluster.hierarchy.linkage(method=“ward”)* (Virtanen *et al.* 2020), resulting in three main clusters as shown in Supplementary Fig. S1. The networks in the individual clusters were merged first, in such a way that their individual edge weights were scaled to all have the same median value and then were added up, a similar approach has been applied in Federico *et al.* (2022). After this was performed for all three clusters the process was repeated for the resulting three new gene similarity networks in order to create one single combined gene similarity network (Fig. 1B2), whose values were again scaled to be in (0,1). The final created network consisted of 22 316 nodes and 213 784 257 edges, which corresponds to a network density of 0.86. The prior network is available at 10.5281/zenodo.7334711.

**Figure 2.**
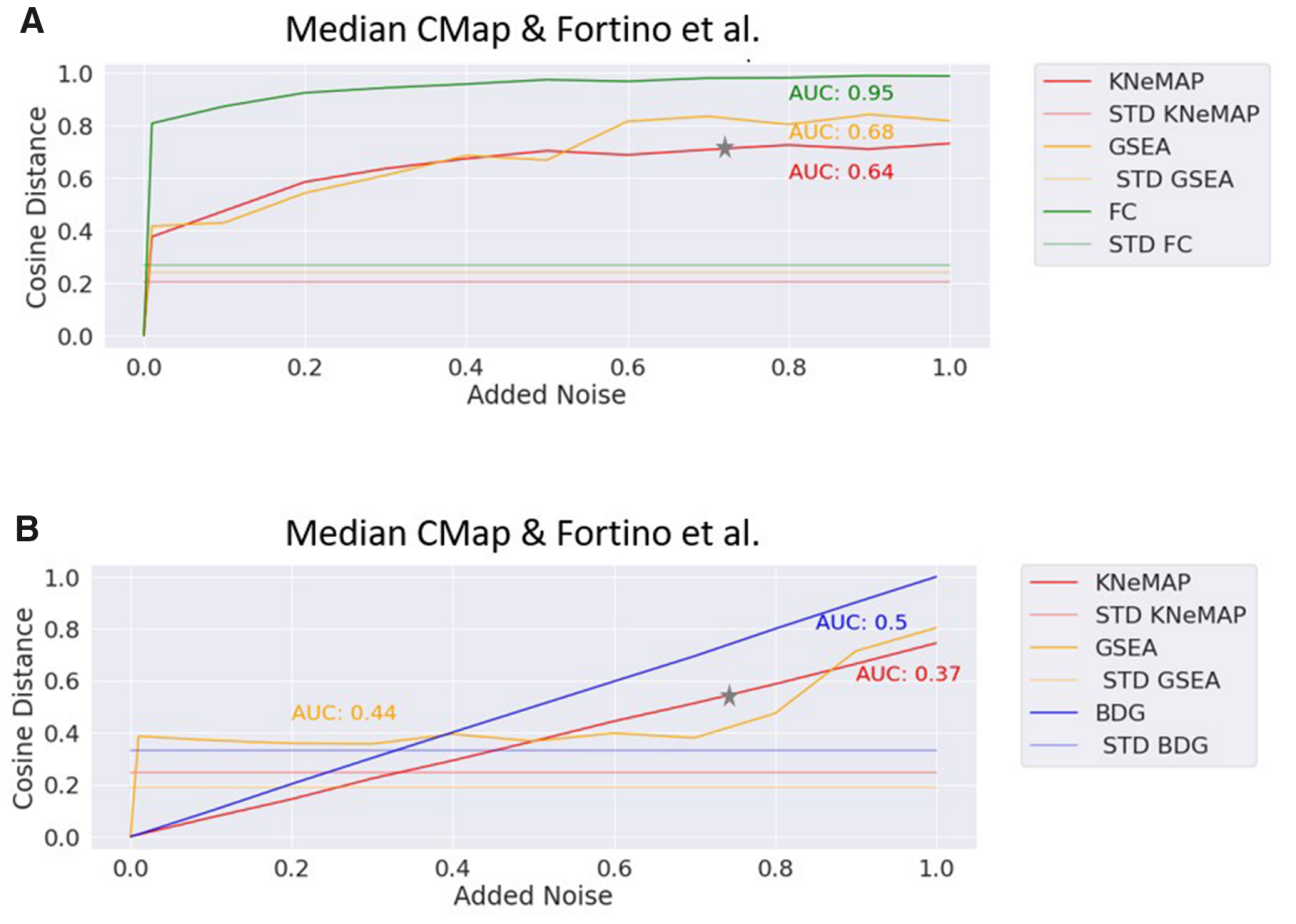
Median cosine distance between KNeMAP, BDG, GSEA, and FC-based vectors with increased levels of added noise to the gene expression values as well as the selected deregulated genes. (A) Shows the median performance across both dataset for increasingly added noise. (B) Shows the median performance across both datasets for increased perturbation noise added to the top 200 selected most deregulated genes. The cosine distance between the vectors was computed from the gene expression data with different noise levels or the set of selected deregulated genes and the baseline (noise = 0). The noise levels are on the *x*-axis, the mean cosine distance on the *y*-axis. The stars are indicators of the KNeMAP line, used to improve inclusivity of the figure.

On the so created weighted gene similarity network community detection was performed (Fig. 1C) with *volta.communities.agglomerative(distance_threshold = 0.5)* (Pavel *et al.* 2021a,b), which performs agglomerative clustering on the networks adjacency matrix using its edge weights (similarities). In order to identify genes that are highly similar in different data layers but not to generate large groups of genes, we aimed at a community distribution of many small-scale communities. In comparison to other community detection algorithms available in VOLTA (Pavel *et al.* 2021a,b), *volta.communities.agglomerative()* showed a partitioning closest to the desired community distribution. The final network partitioning consisted of 1466 communities with a mean size of 15.2 genes per community. The network partitioning is available at https://github.com/fhaive/KNeMAP/tree/main/data.

### 2.2 Feature vector creation

The MOA of a compound can be defined as the list of most deregulated genes (Federico *et al.* 2020, Serra *et al.* 2022b). Thus, KNeMAP compares the drug induced transcriptomic alterations by means of a feature vector, capturing the similarity (gene groups on the prior network) between the most deregulated genes. Additionally, in previous analysis of the CMap dataset, it has been suggested that a subset of affected genes is enough to describe the data instance (biological system + exposure) (Struckmann *et al.* 2021). For each data instance, the genes are sorted by their FCP (±*logFC* * −*log*(*Pval*)) score. The top 100 most positive deregulated genes and the top 100 most negative deregulated genes (Fig. 1D2), which are represented in the created gene similarity network (Fig. 1D1), were selected. Supplementary Figure S4 outlines the correlation and distance between feature vectors for different gene set sizes in combination with the variability of these values. The selected genes were mapped onto the computed communities of the prior network and for each community the fraction of the 200 genes falling into that community were estimated. Based on these fractions, a feature vector for each data instance was generated, where each bit position describes a community and its value indicates the distribution of most deregulated genes across them (Fig. 1D3). The script to compute the vectors is available at https://github.com/fhaive/KNeMAP.

### 2.3 Similarity of the exposures based on the deregulated genes in a binary feature vector

To compare the KNeMAP method, to a commonly used gene-based method (Scala *et al.* 2018, Kinaret *et al.* 2020a, Saarimäki *et al.* 2020, Kinaret *et al.* 2021, Serra *et al.* 2022a), a binary gene vector (BDG) for each data instance was created. To create this vector, the same 200 genes for each instance, as used in the KNeMAP feature vector, were selected. In a gene wide vector (11 868 genes were measured) a value of 1 was set if the corresponding gene at this position is in the set of 200 most deregulated genes of that specific data instance, else a value of 0 was set.

### 2.4 Similarity of the exposures based on the FCP values in a FCP feature vector

We also compared KNeMAP to a vector making use of all gene FCP values of all common measured genes. For each compound exposure on each system, the gene FCP values were collected into a feature vector (FC). A clustermap (Supplementary Fig. S2A), indicating similarities between sample pairs was computed with seaborns (Waskom *et al.* 2018) *clustermap(method=“ward,” metric=“euclidean”).* In addition, the Pearson correlation between all pairwise samples of two biological systems were computed and are displayed in Supplementary Fig. S2B. These two plots show the correlation between instances based on the gene expression fold changes.

### 2.5 Similarity of the exposures based on the GSEA values in a GSEA feature vector

As a third comparison we selected a GSEA (Subramanian *et al.* 2005)-based comparison for KNeMAP, as a more complex and computationally expensive methodology. This approach is in accordance with the method selected by Iorio *et al.* (2010), who used this metric to compute distances between compounds on the CMap dataset. Since KNeMAP, FCP, and BDG are all vectors to describe the alteration profile of a compound on a specific biological system, we computed a GSEA-based vector to describe a compound exposure. For each compound the same top 200 most deregulated genes were selected and used in a GSEA to map against the ranked gene lists (by their FCP) of all the other compounds in a biological system. The GSEA was computed with the blitzGSEA python package (Lachmann *et al.* 2022). The enrichment *P*-values were used to create a feature vector that describes the enrichment of a compound with respect to all other compounds exposed on the same biological system.

### 2.6 Method comparison

#### 2.6.1 Comparing compound similarities to prior knowledge

To evaluate KNeMAP’s performance to other methods, we compared the numerical correlations and similarities based on their distributions as well as with respect to both functional and structural prior knowledge. In addition, we investigated how susceptible to added noise the four methods are. A comparison between KNeMAP, the BDG vectors, the GSEA vectors as well as the FC vectors was performed. The pairwise Pearson correlations and Cosine distances on all three biological systems were computed and their distributions set side by side.

In addition, we compared the four methods based on their ability to identify functional similar compounds. Since the biological system can have a strong impact on the gene expression profiles (Mullard 2018), we focused on identifying similarities on the same biological system rather than between them in order to minimize system dependent biases towards our validation. Our method validation is based on the assumption that drugs with a similar effect should be more similar in their feature vectors than other drugs on the same biological system. In order to describe compound similarity we retrieved ATC (Anatomical Therapeutic Chemical) codes, where possible for compounds in the CMap dataset. ATC codes are unique identifiers assigned to a drug, which is based on the organ it affects as well as how it works. Where the first level describes its anatomical group, the second a drugs therapeutic group, the third level its pharmacological group, the fourth a drug’s chemical group and the last level its chemical substance (https://www.whocc.no/atc_ddd_index/). For 312 drugs respective ATC codes could be retrieved (Supplementary File S1). We used the Pearson correlation to compare the two vectors, as suggested by (Struckmann *et al.* 2021), where it was shown to be the highest performing metrics (out of 26) on the L1000 datasets (CMap 2) (Subramanian *et al.* 2017) in identifying the same chemical across different exposures, which vary in system exposed on or dosage used. To adjust the method to our data, where we only have one exposure of a compound for each biological system, we used the ATC classes to group compounds together. For each compound, c, the other compounds were ranked by their similarity to c, based on the four different feature vectors (KNeMAP, BDG, GSEA, and FC), and the top x (ranging from 1 to the number of compounds for which an ATC code could be retrieved) compounds were selected. Then it was counted how often compounds with the same ATC Code Class (Level 3) were in the top x. This value was divided by the total number of the ATC class in the dataset, in order to limit biases through more represented ATC classes. For each x all values for each c were summed up and displayed in Supplementary Fig. S5. To compare how the methods performed, not only for a specific biological system, we computed the mean for each method across all three biological systems. This allows us to evaluate which method shows the “best” performance on average. The average performance is displayed in Supplementary Fig. S5D. The best performing method is determined by comparing their area under the curve (AUC) scores, where the highest AUC score indicates the best performance. For the NANOSOLUTION data the same metric was performed, however instead of using ATC codes, the core material as well as the ENM shape were used as shown in Supplementary Fig. S9.

In addition, we computed the similarity (based on KNeMAP, the BDG vectors, the GSEA vectors, and the FC vectors) between each compound pair, ranked these pairs based on their similarities and compared the rankings to a similarity computed from the chemical structures. We retrieved the (canonical) SMILES for all CMap compounds, where available, from PubChem (Kim *et al.* 2019, Sayers *et al.* 2022). For each compound pair, in the CMap dataset, (for 450 compounds SMILES were available) the Levenshtein distance, which is the minimum number of character edits needed to make two strings identical (Miller *et al.* 2009), was calculated and the compound pairs were ranked accordingly. This ranking was used as a reference ranking to which the KNeMAP, BDG, GSEA, and FC-based similarity rankings are compared to. Between KNeMAP, BDG, GSEA, and FC, we computed the cosine distance for all compound pairs. Only compounds that had an associated SMILES were considered. These pairs were ranked on their cosine distance. For each method we selected the top x (2–200) compound pairs and computed the rank difference between its rank and the SMILES-based rank. The mean of these values was computed and the results are plotted in Supplementary Fig. S6, the curves are compared by means of their AUC of which a lower value indicates more agreement with the SMILE-based ranking. In addition, we computed the jaccard index based on the top x (1–1000) pairs and compared the performance of all four methods via their AUC scores, of which a high AUC indicates an overall higher jaccard index (Supplementary Fig. S6). This allows us to evaluate the compound pair similarities against a biological system and exposure indifferent factor, the compound structure. To compare how the methods fare not only for a specific biological system, we computed the mean for each method across all three biological systems. This allows us to evaluate which method shows the “best” performance on average. The average performance is displayed in Supplementary Figs S6D and S7D. In addition, we also computed the rank difference for each method’s top 20 compound pairs with the SMILES-based ranking. The density plots of these values, for each biological system, are displayed in Supplementary Fig. S8. For the Fortino *et al.* data, instead of SMILES, functional descriptors of the ENMs, as downloaded from (https://github.com/fhaive/metanalysis_toxicogenomic_data/) were used. Only descriptors available for all ENMs were considered and the cosine distance was estimated between each ENMs descriptor vector of which their pairwise ranks were used the same way as the chemical SMILE-based ranks.

#### 2.6.2 Comparing the impact of added noise between the methods

To investigate how the three different methods are reacting to added noise to the data, two different experiments were performed. First different variations of noise were directly added to the batch corrected gene expression data, from which the ±*logFC* * −*log*(*Pval*) (FCP) scores, as described previously, were calculated. Noise was added per sample, drawn from a Gaussian distribution with mean = 0 and standard deviation levels of 0.01, 0.1, 0.2, 0.3, 0.4, 0.5, 0.6, 0.7, 0.8, 0.9, and 1. For each noise level the KNeMAP, GSEA, and FC vectors, as described previously, were calculated. For each compound, its cosine distance between the added noise levels and the baseline (no noise added to the gene expression vector) was estimated. The mean cosine distance for each noise level across all compounds of a biological system were calculated, together with the average standard deviation (change) across the noise levels, which provides an indication on how much the cosine distance is affected by increasing noise. The cosine distance instead of the Pearson correlation was selected, since we wanted to measure the effect (distance) the different noise levels have with respect to the baseline (noise = 0). For the second experiment the selected 200 most deregulated genes were permuted. Each gene in the selected 200 genes, with a probability of 0.01, 0.1, 0.2, 0.3, 0.4, 0.5, 0.6, 0.7, 0.8, 0.9, and 1 was replaced by another random selected gene from the whole list of measured genes. From this the KNeMAP, GSEA, and BDG vectors were estimated and the cosine distance to their baseline vectors (noise 0) calculated as described in the previous experiment. The results are displayed in Fig. 2, Supplementary Figs S13 and S17.

### 2.7 Stability of KNeMAP vector across different biological systems

To investigate the stability of KNeMAP with respect to differences in steady state gene expressions between different biological systems, we compared the KNeMAP fingerprints computed on different sets of genes. Exposures on different biological systems are known to be different, which partially is caused by the differences in steady state gene expression. To showcase that KNeMAP is robust to such change, we compute the KNeMAP fingerprints based only on the genes that are not differentially expressed as well as only differential expressed genes between the control samples of the individual CMap cell systems. A gene was differentially expressed, if it was classified as differentially expressed between at least one cell line pair. Differential expression analysis was performed with limma() (Ritchie *et al.* 2015), as already described in the Supplementary Materials for the pre-processing of the CMap dataset. We then computed the cosine distance between each compound pair on a biological system for both types of vectors and then estimated the difference in cosine distance for each compound pair. The distribution of differences is plotted in Supplementary Fig. S21, showing that there is a minimal change in pairwise distance between the fingerprints computed based on the complete gene vector or only when taking stable genes between all biological systems into account, due to the independence and multi-dimension of the prior gene–gene network.

### 2.8 Individual analysis of the CMap and Fortino *et al.* dataset

Transcriptomics profiles alterations induced by compound exposure under different experimental conditions (e.g. biological systems, exposure time) can vary strongly (Kinaret *et al.* 2017, Fortino *et al.* 2022). In addition data biases can be present, e.g. due to technical differences, batch effects or to underlying differences in the biological systems (Supplementary Fig. S3) (Federico *et al.* 2020, Serra *et al.* 2020). Therefore we decided to analyze, for the CMap dataset, the three different biological systems independently from each other and merge their results in order to identify similarities between the systems. Analyzing the biological systems independently, allows us to compare the MOA of the exposures detached from the underlying data and in result minimizes data and system related biases, which has been suggested to be an issue of the CMap dataset (Lim and Pavlidis 2021). We performed the same analysis pipeline for the two biological systems available in the Fortino *et al.* dataset. The analysis methodology is described in detail in the Supplementary Materials (Methods—Comparison of the Biological Systems).

### 2.9 Comparative analysis between the CMAP and Fortino *et al.* dataset

To showcase the capability of KNeMAP to compare transcriptomic alteration profiles across datasets, we performed a comparative analysis between the transcriptomic profiles induced by the ENM and drug exposures. Thus, for each ENM in the Fortino *et al.* data we retrieved the most similar drug in the CMap dataset. For each exposure instance between the Fortino *et al.* data and the CMap data we computed the cosine distance between their KNeMAP feature vectors, then ranked the drugs according to their similarity to a nanomaterial exposure. For each nanomaterial–drug pair the mean rank was estimated and the highest ranked drug was selected for the ENM. It is important to note, that when the same prior network is used, KNeMAP offers the possibility to compare different datasets without the need to recompute or adjust the computed feature vectors.

## 3 Results

We developed KNeMAP, a novel methodology for comparison of transcriptomic profiles. We showcased the effectiveness of our method by analyzing the Connectivity Map (CMap) dataset (Lamb *et al.* 2006) and the Fortino *et al.* (2022) dataset. The CMap dataset is a popular reference database for drug-induced expression profiles and combines chemical exposures over three cell lines of which 11 868 genes are measured across all three biological systems. The diversity of CMap makes it a suitable dataset for the identification of groups of chemicals that act similarly on different biological systems, which are challenging to identify with traditional gene-based methods. In Supplementary Fig. S3 the steady state gene expression profiles of the three different cell lines are outlined, which are very different. The Fortino *et al.* dataset comprises transcriptomic profiles of different nanomaterials exposed on two human cell lines (THP-1 and BEAS-2B). The materials vary in core material as well as in their surface chemistry. We evaluated KNeMAP against three existing methods: BDG, GSEA, and FC, by comparing the similarity of transcriptomic profiles calculated with the three methods against similarities computed with independent data layers such as the chemical structure and functional knowledge.

### 3.1 KNeMAP-based similarities better resemble those computed from prior knowledge

To evaluate the performance of KNeMAP, we investigated how it performs with respect to prior knowledge. Since prior knowledge was not equally available for all compounds, these metrics were only computed for compounds where the considered prior knowledge was available. To evaluate the method's capability in identifying structurally similar CMap compounds, pairwise compound similarities were estimated and their rankings compared to compound pair rankings based on KNeMAP, BDG, GSEA, and FC-based vectors. Supplementary Figures S6 and S7 showcase the improvement in agreement to the structural-based ranking for KNeMAP. While differences in performance between the biological systems could be observed. On average (Supplementary Figs S6D and 7D) KNeMAP is in more agreement with the structural-based ranking, which is indicated by lower AUC values (the difference to a structural-based ranking is measured) in Supplementary Fig. S6, a higher AUC values in Supplementary Fig. S6 and a shift of the distribution to the left in Supplementary Fig. S8.

Supplementary Figure S5, showcases the performance of KNeMAP in comparison to BDG, GSEA, and FC in identifying functionally similar CMap compounds. Functional similarity of compounds was determined based on their ATC level 3 codes. However, on average the performance across all three systems is very similar between the methods. For the Fortino *et al.* data, KNeMAP outperforms the other methods on average on identifying ENMs with the same shape (Supplementary Fig. S9E), while GSEA and FC show stronger performance in identifying ENMs based on their core-material (Supplementary Fig. S9F). This suggests that it is advisable to select a metric based on the task to be performed and data quality available. While for the molecular descriptor-based ranking KNeMAP was outperformed by FC for the difference in rankings and BDG for the jaccard index, it performed second best for both methods, overall showing the most stable performance, as displayed in Supplementary Fig. S10.

### 3.2 KNeMAP reduces the noise associated to transcriptomic studies and improves the retrieval of similarity patterns

To show the improvement on the overall comparability of the investigated datasets and to investigate the impact KNeMAP has on the overall similarity distributions, we compared the within dataset distance and correlation by means of the Pearson correlation and cosine distance.

When comparing the Pearson correlation and cosine distance distribution values for each compound pair on each biological system (Supplementary Figs S11 and S12) for the FC vectors, the BDG vectors, the GSEA vectors and KNeMAP, it can be observed that while the BDG and FC-based values show a similar narrow peaked distribution at 0 and 1 respectively, KNeMAP and GSEA yield a broader distribution shifted to the right and left respectively, while GSEA shows a strong difference in shape between the data-sets in contrast to the other three methods. This indicates a shift in similarity/correlation between the exposures, which is not observable based on traditional methods, making this previously difficult dataset easier to analyze and to identify similarities between exposures by reducing the noisy peak observable with the other two methods.

As shown in Fig. 2, KNeMAP is less impacted on average by increasingly added noise to the gene expression values in comparison to the FC and GSEA-based cosine distance. The same applies to KNeMAP in comparison to BDG and GSEA when impacting the selected deregulated genes, which is indicated by its overall lower AUC score.

In Supplementary Figs S14, S15, S18, and S19 the plots are shown for selected compounds, Supplementary Figs S13 and S17 show the performance for each individual biological system as well as the median for each dataset and Supplementary Figs S16 and S20 showcases the standard deviation distribution for each biological system for the cosine distance against its baseline (noise = 0). Next to the overall better AUC scores that KNeMAP achieves (Fig. 2), it can be observed that KNeMAP, FC, and BDG are relatively stable across all five biological systems with respect to their AUC scores, while the performance of GSEA varies strongly across biological systems (Supplementary Figs S13 and S17).

### 3.3 Comparison of transcriptomic profiles across different cell lines identifies compounds with a system dependent similar mechanism of action

Through the clustering of the compounds (Fig. 1E) across the three different biological systems of the CMap dataset, based on KNeMAP, we were able to identify a set of 38 drugs (Supplementary Fig. S22) that behave similarly when exposed on the same biological system (Fig. 1F). From now on, we consider these 38 chemicals during further analysis. Given the low correlation between the individual MOAs (Supplementary Fig. S2A), we hypothesize that these drugs might have different responses in different systems, while showing similarities when exposed to the same cancer cell lines. It is often observed that molecular heterogeneity across cancer cell lines causes differences in response to the same drug, possibly offering a biological explanation to the observed phenomenon (Dagogo-Jack and Shaw 2018). When clustering the individual treatments, it is apparent how they group by the exposed biological system (Supplementary Fig. S23), rather than by drug. Therefore, we investigated possible characteristics of the 38 drugs that would be responsible for their similar behavior. When addressing their therapeutic indications, 33% were antimicrobial drugs, 15% cardiac glycosides (antiarrhythmic agent), 10% hsp90 inhibitors, and 10% antipsychotic (Supplementary Fig. S26). Although all these drug classes have been already repurposed for various cancer treatments, no specific primary molecular target or pathway could justify their similar activity. Therefore, we hypothesized that the chemical structure may be responsible for the observed phenomenon. Through scaffold analysis (Supplementary Table S2) we were able to identify high level scaffolds statistically enriched in this set of drugs (Supplementary Fig. S27) that can interact with membranes, cytoskeleton and alter the redox state. All these targets are very sensible in cancer cell lines, and when targeted they ultimately induce a cytostatic or cytotoxic effect. We further explored the structure information to identify other compounds that may show the same or similar behavior when exposed on the same biological systems (Supplementary Table S3).

To showcase the functionality of KNeMAP, we also applied this approach to a set of ENMs exposed to two different cell lines. As in the first case study, our approach was able to highlight a cluster of hazardous nanoparticles (gold and quantum dots with various functionalizations) with peculiar optical and electronic properties (Supplementary Fig. S28). It is known that physicochemical characteristics of nanomaterials affect the induced biological response, possibly explaining the observed similarities across cell lines (Liu *et al.* 2006, Ellis *et al.* 2020). A detailed description of the analysis results and the identified drugs can be found in the Supplementary Materials (Results—Comparison of Transcriptomic Profiles Across Different Cell Lines Identifies Drugs with a System Dependent Similar Mechanism of Action and Description of the Identified Nanomaterials).

### 3.4 Identifying drugs and nanomaterials with a similar mechanism of action

Through the comparison of the KNeMAP fingerprints of the Fortino *et al.* data with the CMap data, we identified for each nanomaterial the chemical compound with the most similar MOA across all biological systems. All identified pairs are listed in Supplementary Table S5 and detailed descriptions of selected pairs are provided in the Supplementary Materials (Results—Identifying the Most Similar Chemical for each Nanomaterial Based on their Mechanism of Action). For example a copper oxide nanomaterial was found to act similar to Lycorine and both have been shown to affect acetylcholinesterase and in result the nervous system (Sezer Tuncsoy *et al.* 2019, Kola *et al.* 2023).

## 4 Discussion/conclusions

We propose KNeMAP as a new knowledge-driven method to compare transcriptomic profiles. In comparison to other methods, which focus on individual genes, KNeMAP groups genes into a “similarity group,” which allows to compare expression profiles in a higher-level manner than when comparing genes individually. We showed that a network mapping-based approach is able to identify similar compounds in higher agreement with functional as well as structural prior knowledge, when compared to the BDG, GSEA, and FC methods. In addition, it is able to reduce the observable noise in the data, which makes the dataset easier to analyze and allows it to identify patterns. KNeMAP can be especially suitable for datasets where data from different systems and with different exposure parameters are compared. In this work, the KNeMAP was applied on the CMap (Lamb *et al.* 2006) dataset as well as the Fortino *et al.* dataset (Gallud *et al.* 2020, Kinaret *et al.* 2021) and we were able to identify a set of compounds that always show a similar response between each other on the same biological system, even though their response may vary across biological systems. While the identified CMap compounds have different therapeutic uses and molecular targets they all have been linked to similar effects on cancer, and have often been repurposed for oncological treatments. Since they do not share most of the molecular mechanism, a more traditional comparison between differentially expressed genes would have not identified this commonality. The underlying differences of the biological systems can explain the differences in expression patterns between the biological systems for similar compounds, suggesting that these compounds affect the cancer cells differently but always in a similar manner between each other (on the same biological system). In order to make statements about the comparability or the behavior of these compounds on non-cancer related biological systems, further analysis needs to be done, showcasing again how important it is to understand the comparability between biological systems with respect to chemical safety assessment. Moreover, when compared with three different gene focused approaches, KNeMAP is able to identify similarities between compounds with higher agreement to functional as well as structural information. When comparing transcriptomic experiments, one limitation is given by the fact that the same molecules (e.g. genes) need to be profiled. However, different experiments are often performed on different platforms, with only partially overlapping probes/genes. The KNeMAP approach can be further exploited in this case and be used to compare the datasets since thanks to the fact that genes can be grouped into communities, no one-to-one mapping between the genes is required. We showcase this by comparing the CMap dataset with the Fortino *et al.* dataset by identifying for each nanomaterial the drug with the most similar MOA across all biological systems. Furthermore, KNeMAP is highly flexible with respect to what prior data is used to construct the network, so can, e.g. only a single data layer (e.g. pathways, GO) be used or a subset of layers, as well as to the size of gene communities to be detected (based on the algorithm chosen). This allows a “stricter” or “looser” view on gene similarity as needed based on the data or study. In conclusion KNeMAP is a generic approach, that can be customized with respect to prior information and gene clusters used, to compare noisy transcriptomic datasets.

## Supplementary Material

btad341_Supplementary_Data

## References

[btad341-B1] Dagogo-Jack I , Shaw AT. Tumour heterogeneity and resistance to cancer therapies. Nat Rev Clin Oncol 2018;15:81–94. 29115304 10.1038/nrclinonc.2017.166

[btad341-B2] Ellis GA , Dean SN, Walper SA et al Quantum dots and gold nanoparticles as scaffolds for enzymatic enhancement: recent advances and the influence of nanoparticle size. Catalysts 2020;10:83.

[btad341-B3] Federico A , Serra A, Ha MK et al Transcriptomics in toxicogenomics, part II: preprocessing and differential expression analysis for high quality data. Nanomaterials (Basel) 2020;10:903. 32397130 10.3390/nano10050903 PMC7279140

[btad341-B4] Federico A , Fratello M, Scala G et al Integrated network pharmacology approach for drug combination discovery: a multi-cancer case study. Cancers (Basel) 2022;14:2043. 35454948 10.3390/cancers14082043 PMC9028433

[btad341-B5] Fortino V , Kinaret PAS, Fratello M et al Biomarkers of nanomaterials hazard from multi-layer data. Nat Commun 2022;13:3798. 35778420 10.1038/s41467-022-31609-5 PMC9249793

[btad341-B6] Fratello M , Cattelani L, Federico L et al Unsupervised algorithms for microarray sample stratification. Methods Mol Biol 2022;2401:121–46. 34902126 10.1007/978-1-0716-1839-4_9

[btad341-B7] Freytag S , Gagnon-Bartsch J, Speed TP et al Systematic noise degrades gene co-expression signals but can be corrected. BMC Bioinformatics 2015;16:309. 26403471 10.1186/s12859-015-0745-3 PMC4583191

[btad341-B8] Gallud A , Delaval M, Kinaret P et al Multiparametric profiling of engineered nanomaterials: unmasking the surface coating effect. Adv Sci (Weinh) 2020;7:2002221. 33240770 10.1002/advs.202002221 PMC7675037

[btad341-B9] Gao S , Han L, Luo D et al Modeling drug mechanism of action with large scale gene-expression profiles using GPAR, an artificial intelligence platform. BMC Bioinformatics 2021;22:17. 33413089 10.1186/s12859-020-03915-6 PMC7788535

[btad341-B10] Iorio F , Isacchi A, di Bernardo D et al Identification of small molecules enhancing autophagic function from drug network analysis. Autophagy 2010;6:1204–5. 20930556 10.1073/pnas.1000138107

[btad341-B11] Kim S , Chen J, Cheng T et al PubChem 2019 update: improved access to chemical data. Nucleic Acids Res 2019;47:D1102–9. 30371825 10.1093/nar/gky1033 PMC6324075

[btad341-B12] Kinaret P , Marwah V, Fortino V et al Network analysis reveals similar transcriptomic responses to intrinsic properties of carbon nanomaterials in vitro and in vivo. ACS Nano 2017;11:3786–96. 28380293 10.1021/acsnano.6b08650

[btad341-B13] Kinaret PAS , Scala G, Federico A et al Carbon nanomaterials promote M1/M2 macrophage activation. Small2020a;16:e1907609. 32250056 10.1002/smll.201907609

[btad341-B14] Kinaret PAS , Serra A, Federico A et al Transcriptomics in toxicogenomics, part I: experimental design, technologies, publicly available data, and regulatory aspects. Nanomaterials (Basel) 2020b;10:750. 32326418 10.3390/nano10040750 PMC7221878

[btad341-B15] Kinaret PAS , Ndika J, Ilves M et al Toxicogenomic profiling of 28 nanomaterials in mouse airways. Adv Sci (Weinh) 2021;8:2004588. 34026454 10.1002/advs.202004588 PMC8132046

[btad341-B16] Kola A et al A comparative study between lycorine and galantamine abilities to interact with AMYLOID β and reduce in vitro neurotoxicity. Int J Mol Sci 2023;24:2500. 36768823 10.3390/ijms24032500 PMC9916559

[btad341-B17] Lachmann A , Xie Z, Ma'ayan A et al blitzGSEA: efficient computation of gene set enrichment analysis through gamma distribution approximation. Bioinformatics 2022;38:2356–7. 35143610 10.1093/bioinformatics/btac076 PMC9004650

[btad341-B18] Lamb J , Crawford ED, Peck D et al The connectivity map: using gene-expression signatures to connect small molecules, genes, and disease. Science 2006;313:1929–35. 17008526 10.1126/science.1132939

[btad341-B19] Lim N , Pavlidis P. Evaluation of connectivity map shows limited reproducibility in drug repositioning. Sci Rep 2021;11:17624. 34475469 10.1038/s41598-021-97005-z PMC8413422

[btad341-B20] Liu N , Prall BS, Klimov VI et al Hybrid gold/silica/nanocrystal-quantum-dot superstructures: synthesis and analysis of semiconductor-metal interactions. J Am Chem Soc 2006;128:15362–3. 17131988 10.1021/ja0660296

[btad341-B21] Marwah VS , Scala G, Kinaret PAS et al eUTOPIA: solUTion for Omics data PreprocessIng and Analysis. Source Code Biol Med 2019;14:1. 30728855 10.1186/s13029-019-0071-7 PMC6352382

[btad341-B22] Miller FP , Vandome A, McBrewster J. Levenshtein distance: information theory, computer science, string (computer Science), String metric, Damerau? Levenshtein distance, Spell checker, Hamming distance. Alpha Press, Orlando, 2009.

[btad341-B23] Mitra S , Saha S, Hasanuzzaman M et al Multi-view clustering for multi-omics data using unified embedding. Sci Rep 2020;10:13654. 32788601 10.1038/s41598-020-70229-1 PMC7423957

[btad341-B24] Mullard A. Can you trust your cancer cell lines? Nat Rev Drug Discov 2018;17:613. 10.1038/nrd.2018.154 30160254

[btad341-B25] Pavel A , Del Giudice G, Federico A et al Integrated network analysis reveals new genes suggesting COVID-19 chronic effects and treatment. Brief Bioinf2021a;22:1430–41. 10.1093/bib/bbaa417 PMC7929418 33569598

[btad341-B26] Pavel A , Federico A, Del Giudice G et al VOLTA: adVanced mOLecular neTwork Analysis. Bioinformatics2021b;37:4587–8. 34498028 10.1093/bioinformatics/btab642 PMC8687180

[btad341-B27] Pavel A , Saarimäki LA, Möbus L et al The potential of a data centred approach & knowledge graph data representation in chemical safety and drug design. Comput Struct Biotechnol J 2022;20:4837–49. 36147662 10.1016/j.csbj.2022.08.061 PMC9464643

[btad341-B28] Rappoport N , Shamir R. Multi-omic and multi-view clustering algorithms: review and cancer benchmark. Nucleic Acids Res 2018;46:10546–62. 30295871 10.1093/nar/gky889 PMC6237755

[btad341-B29] Raser JM , O'Shea EK. Noise in gene expression: origins, consequences, and control. Science 2005;309:2010–3. 16179466 10.1126/science.1105891 PMC1360161

[btad341-B30] Ritchie ME , Phipson B, Wu D et al limma powers differential expression analyses for RNA-sequencing and microarray studies. Nucleic Acids Res 2015;43:e47. 25605792 10.1093/nar/gkv007 PMC4402510

[btad341-B31] Saarimäki LA , Kinaret PA, Scala G et al Toxicogenomics analysis of dynamic dose-response in macrophages highlights molecular alterations relevant for multi-walled carbon nanotube-induced lung fibrosis. NanoImpact 2020;20:100274.

[btad341-B32] Sayers EW , Bolton EE, Brister JR et al Database resources of the national center for biotechnology information. Nucleic Acids Res 2022;50:D20–6. 34850941 10.1093/nar/gkab1112 PMC8728269

[btad341-B33] Scala G , Kinaret P, Marwah V et al Multi-omics analysis of ten carbon nanomaterials effects highlights cell type specific patterns of molecular regulation and adaptation. NanoImpact 2018;11:99–108. 32140619 10.1016/j.impact.2018.05.003 PMC7043328

[btad341-B34] Serra A , Fratello M, Fortino V et al MVDA: a multi-view genomic data integration methodology. BMC Bioinformatics 2015;16:261. 26283178 10.1186/s12859-015-0680-3 PMC4539887

[btad341-B35] Serra A , Coretto P, Fratello M et al Robust and sparse correlation matrix estimation for the analysis of high-dimensional genomics data. Bioinformatics 2018;34:625–34. 29040390 10.1093/bioinformatics/btx642

[btad341-B36] Serra A , Fratello M, Cattelani L et al Transcriptomics in toxicogenomics, part III: data modelling for risk assessment. Nanomaterials (Basel) 2020a;10:708. 32276469 10.3390/nano10040708 PMC7221955

[btad341-B37] Serra A , del Giudice G, Kinaret PAS et al Characterization of ENM dynamic dose-dependent MOA in lung with respect to immune cells infiltration. Nanomaterials (Basel) 2022b;12:2031. 35745370 10.3390/nano12122031 PMC9228743

[btad341-B38] Serra A , Saarimäki LA, Pavel A et al Nextcast: a software suite to analyse and model toxicogenomics data. Comput Struct Biotechnol J 2022;20:1413–26. 35386103 10.1016/j.csbj.2022.03.014 PMC8956870

[btad341-B39] Sezer Tuncsoy B , Tuncsoy M, Gomes T et al Effects of copper oxide nanoparticles on tissue accumulation and antioxidant enzymes of Galleria mellonella L. Bull Environ Contam Toxicol 2019;102:341–6. 30600390 10.1007/s00128-018-2529-8

[btad341-B40] Struckmann S , Ernst M, Fischer S et al Scoring functions for drug-effect similarity. Brief Bioinf 2021;22:bbaa072. 10.1093/bib/bbaa072 PMC8138836 32484516

[btad341-B41] Subramanian A , Tamayo P, Mootha VK et al Gene set enrichment analysis: a knowledge-based approach for interpreting genome-wide expression profiles. Proc Natl Acad Sci USA 2005;102:15545–50. 16199517 10.1073/pnas.0506580102 PMC1239896

[btad341-B42] Subramanian A , Narayan R, Corsello SM et al A next generation connectivity map: L1000 platform and the first 1,000,000 profiles. Cell 2017;171:1437–52.e17. 29195078 10.1016/j.cell.2017.10.049 PMC5990023

[btad341-B43] The Gene Ontology Consortium. The gene ontology resource: enriching a GOld mine. Nucleic Acids Res 2021;49:D325–34. 33290552 10.1093/nar/gkaa1113 PMC7779012

[btad341-B44] Virtanen P , Gommers R, Oliphant TE et al; SciPy 1.0 Contributors. SciPy 1.0: fundamental algorithms for scientific computing in Python. Nat Methods 2020;17:261–72. 32015543 10.1038/s41592-019-0686-2 PMC7056644

[btad341-B45] Waskom M , Botvinnik O, O'Kane D et al mwaskom/seaborn: v0.9.0 (July 2018). [Computer software]. *Zenodo*. 2018. 10.5281/zenodo.1313201.

