# Supplementary material for: KNeMAP: a network mapping approach for knowledge-driven comparison of transcriptomic profiles
Source: Bioinformatics. 2023 May 24;39(6):btad341. doi: 10.1093/bioinformatics/btad341 (PMC10243850; doi:10.1093/bioinformatics/btad341)
Supplement: btad341_Supplementary_Data [file btad341_supplementary_data.zip › KNEMAP Supplementary.pdf]

KNeMAP: A Network Mapping Approach for Knowledge-driven Comparison of Transcriptomic Profiles

Alisa Pavel[1,2,3], Giusy del Giudice[1,2,3], Michele Fratello[1,2,3], Leo Ghemtio[4], Antonio Di Lieto[5], Jari Yli-Kauhaluoma[4], Henri Xhaard[4], Antonio Federico[1,2,3,6], Angela Serra[1,2,3,6] and Dario Greco[1,2,3,7,8*]

[1] Faculty of Medicine and Health Technology, Tampere University, Tampere, Finland.
[2] BioMediTech Institute, Tampere University, Tampere, Finland
[3] Finnish Hub for Development and Validation of Integrated Approaches (FHAIVE), Tampere, Finland
[4] Drug Research Program, Division of Pharmaceutical Biosciences, Faculty of Pharmacy, University of Helsinki, Helsinki, Finland
[5] Mental Health Services, Landspitali University Hospital, 101 Reykjavik, Iceland
[6] Tampere Institute for Advanced Study, Tampere, Finland
[7] Institute of Biotechnology, University of Helsinki, Helsinki, Finland
[8] Division of Pharmaceutical Biosciences, Faculty of Pharmacy, University of Helsinki, Helsinki, Finland

* Corresponding author: dario.greco@tuni.fi

Methods

Collection of Expression Data and Pre-Processing

A total of 6,029 Affymetrix GeneChip raw data files (.CEL files) belonging to the HTHG-U133A chipset were collected from the Connectivity Map (CMap) (Lamb *et al.*, 2006) website (CMap 1 data set).

The CMap data set contains samples from three different Affymetrix platforms as listed in table 1. To avoid batch effects, as a result of the different platforms during pre-processing, only samples from platform HT_HG-U133A were included in the analysis, since it contains the highest number of samples. The samples available for the HT_HG-U133A platform contained data for three human cell lines, PC3, MCF7 and HL60.

	HG-U133A	HT_HG-U133A	HT_HG-U133A_EA
Control	133	787	36
Treatment	625	5242	184
Sum	758	6029	220

Table 1: Number of samples for each platform.

Raw data were imported in R by using the *ReadAffy()* function from the affy library (Gautier *et al.*, 2004). First, the sample outliers were identified by using statistics coming from the RLE, NUSE and RNA Degradation curves. The samples marked as outliers from at least two of the three methods were removed from the analysis. In this way, 104 samples were removed. Expression values were normalized, background-corrected, summarized and log2 transformed by using the *justRMA()* function of the affy package (Gautier *et al.*, 2004). Afterward, the combat method was applied to remove the known batch effect due to the batch id variable present in the dataset.
For each of the drugs tested on all cell lines, a limma based approach was applied to identify the deregulated genes for each drug versus the untreated controls in each cell line. Subsequently, the genes were ranked by $\pm logFC * -log(Pval)$ (FCP), resulting in ordered gene lists having the most up-regulated genes on the top and the most down-regulated genes on the bottom (manuscript figure 1 A). The pre-processed data is available at 10.5281/zenodo.7334711.

To generate the final data set, the intersection of genes present in the three different data sets as well as the intersection of compounds were retrieved, resulting in a final data set of 11,868 genes for 676 compounds exposed on three different biological systems (MCF7, PC3 and HL60).

The Fortino et al. data (Fortino *et al.*, 2022) was downloaded and processed with eUTOPIA (Marwah *et al.*, 2019), as described in (Saarimäki *et al.*, 2021). The final dataset consists out of 31 nanomaterials (ENM), with different core materials and surface chemistries as described in (Gallud *et al.*, 2020; Kinaret *et al.*, 2021), across two different biological systems (THP-1 and BEAS-2B), covering in total 20,865 genes.

Prior Network Creation & Community Detection

Data Collection
Protein family groupings were retrieved from Panther (Thomas *et al.*, 2003; Mi *et al.*, 2021) (downloaded 10/2019), protein paralog information from Ensembl (Howe *et al.*, 2021) (downloaded 10/2019) and homologue gene groups were retrieved from NCBI (Sayers *et al.*, 2022) (downloaded 10/2019). Gene chemical and gene disease associations were retrieved from CTD (Davis *et al.*, 2021) (downloaded 10/2019). Gene molecular function, gene biological process and gene cellular component associations were collected from GO (The Gene Ontology Consortium, 2021) (downloaded 12/2019), while protein protein interaction (PPI) information were collected from HIPPIE (Alanis-Lobato *et al.*, 2017) (downloaded 10/2019), KEGG (Kanehisa and Goto, 2000; Kanehisa *et al.*, 2017) (downloaded 12/2019), HitPredict (López *et al.*, 2015) (downloaded 11/2019) & Reactome (Jassal *et al.*, 2020) (downloaded 01/2010) and gene pathway associations were retrieved from Reactome (Jassal *et al.*, 2020) (downloaded 01/2010), Wikipathway (Martens *et al.*, 2021) (downloaded 01/2020) & KEGG (Kanehisa and Goto, 2000; Kanehisa *et al.*, 2017) (downloaded 12/2019). All genes and gene products were mapped to their corresponding Ensembl Gene IDs as has been described in (A Pavel *et al.*, 2021) and integrated into a Knowledge Graph framework

(Pavel, Saarimäki, *et al.*, 2022), named the Unified Knowledge Space (UKS) as described in (A Pavel *et al.*, 2021; Federico *et al.*, 2022).

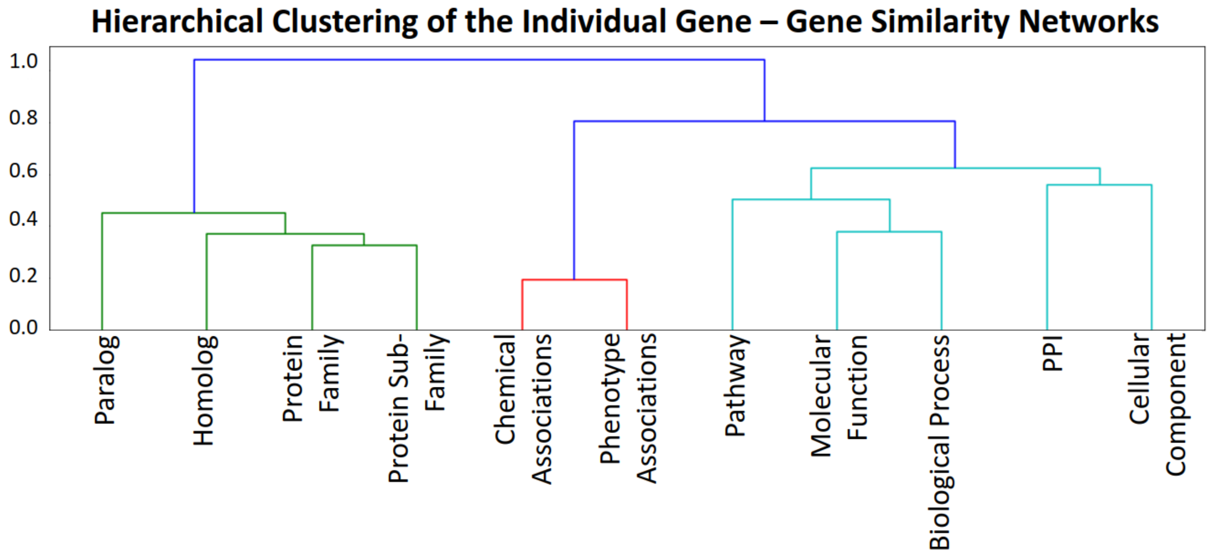

Figure 1: Hierarchical clustering of Individual gene - gene similarity networks, showcasing the three sub-prior networks before merging them into one combined network.

Comparison of the Biological Systems

To investigate how similar the gene expression profiles of the different biological systems are, we computed the discrete fuzzy patterns (dfp) (Díaz *et al.*, 2006; Glez-Peña *et al.*, 2009) profiles of the controls. The dfp method uses all of a gene's expression values (across replicates for a biological system) and fits functions to these values in order to assign a probability score for each expression class (low, mid and high). The expression class is determined for each gene if its probability score is ≥ 0.5.

Clustering & Cluster Similarity

For each vector pair (KNeMAP) on the same system its euclidean, canberra, cosine, manhattan, jensen-shannon and chebyshev distances were calculated with *scipy.spatial.distance() (Virtanen et al., 2020a)*.

For each of these distances 10 clusterings for 4 different clustering algorithms were estimated. For algorithms containing randomness their individual 10 clusterings differ, while for algorithms without randomness 10 times the same clustering is added in order to consider all algorithms as equal. This strategy has been applied in (Alisa Pavel *et al.*, 2021) and is based on the wisdom of crowd principle (Marbach *et al.*, 2012). The applied clustering algorithms are *volta.clustering.hierarchical_clustering(linkage="complete"), volta.clustering.kmediods_clustering(), volta.clustering.affinityPropagation_clustering(), volta.clustering.optics_clustering(*`radius=2`*)*. When applicable the algorithm parameters were tuned with *volta.clustering.multiobjective(min_number_clusters=None, max_number_clusters=None, min_cluster_size=None, max_cluster_size=None, local =True, bet=True, e=None, s=None, cluster_size_distribution=True)*. The parameters tuned were number of clusters for *volta.clustering.hierarchical_clustering(linkage="complete"),*

volta.clustering.kmediods_clustering and *volta.clustering.optics_clustering(*`radius=2)`. In addition the number of neighbors was also optimized for *volta.clustering.optics_clustering(*`radius=2)`. The parameter selection of *volta.clustering.multiobjective(),* was set to find the number of clusters that optimize high within cluster similarity, high between cluster dissimilarity as well as an equal cluster size distribution. Based on the resulting individual clusterings, a consensus clustering was estimated for the CMap dataset with *volta.clustering.consensus_clustering(threshold=0.9, per_node=True, rep=100)* & *volta.clustering.consensus_clustering(threshold=None, per_node=True, rep=100)* for the Fortino et al. dataset, which iteratively computes an agreement matrix between multiple clustering outputs, removes weak edges and computes multiple new clusters on the agreement graph based on the louvain algorithm (Aynaud, 2020; Pavel, Serra, *et al.*, 2022). This is repeated until convergence or the maximum number of iterations has been reached. For more details please refer to the VOLTA manual (Alisa Pavel *et al.*, 2021). A consensus approach based on multiple distances and different clustering algorithms was selected, following the "wisdom of crowds" principle, which suggests that a group may be closer to the unknown truth than a single expert (algorithm, distance) (Marbach *et al.*, 2012). The identified clusters for each cell line were compared in pairs, via a jaccard index to identify cluster pairs between biological systems that contain a high number of shared compounds (manuscript figure 1 F). This allows us to identify a set of compounds, which show similar behavior between each other in each biological system, even though the response may be system dependent.

Description of the Identified Drugs

ATC Codes

When possible the ATC codes for a compound were retrieved from WHO (World Health Organization) (https://www.whocc.no/atc_ddd_index/), however nearly 60% of compounds did not have an assigned ATC code. A complete list of compounds and their mapped ATC codes is provided in supplementary file 1. The distributions of ATC Level 1 and Level 2 were analyzed in the set of identified compounds.

Drug Targets

Drugs were linked to their gene targets via DrugBank (Wishart *et al.*, 2018, 2008), for 12% of compounds no gene targets were available at the time of retrieval. These gene targets were linked to their corresponding Protein Families via the PANTHER (Mi *et al.*, 2021; Thomas *et al.*, 2003) Protein Family Groups. The distributions of protein families of the targets of the set of identified drugs are displayed in figure 25.

Scaffolds

Scaffolds were computed from all compounds for which a SMILES could be retrieved from PubChem (Kim *et al.*, 2019) (450 in total). Scaffolds were computed with rdkit *(rdkit.org)* and scaffoldgraph (Scott and Edith Chan, 2020). Afterwards, a hypergeometric test was performed with *scipy.stats.hypergeom() (Virtanen et al., 2020a)*, where the occurrence of each scaffold in class 1 (drugs of interest) is compared against class 0 (all other drugs). The p-values were corrected with *statsmodels.stats.multitest.multipletests(method="fdr-bh") (Seabold and Perktold, 2010)*, which applies a Benjamini Hochberg correction (Benjamini

and Hochberg, 1995). A p-value cut-off of 0.05 was applied. The enriched scaffolds are displayed in supplementary table 2.

To identify compounds that may show a similar mechanism of action as the identified drugs, the top level scaffolds of the statistical enriched scaffolds were used to identify compounds not in the 676 investigated compounds, but also contain the same or a highly similar structural component. The SMILE of each top-level scaffold (figure 27) (statistically enriched and parent of other statistically enriched scaffolds) was used in a PubChem (Kim *et al.*, 2019) substructure search. The top 3 returned compounds, not contained in the 676 drugs under investigation, were selected (table 3).

Results

CMap Similarity based on Expression Values

After the described pre-processing of the CMap data (main text), for each chemical exposure on each system the gene expression values were collected into a feature vector of length 11 868. A clustermap (figure 2 A), indicating similarities between pairs was computed with seaborns (Waskom *et al.*, 2018) *clustermap(X, method = "ward", metric="euclidean", figsize=(40,40)).* In addition the Pearson correlation between all pairwise samples of two systems were computed and are displayed in figure 2 B, indicating a correlation of close to 0 for most of the pairs.
These results showcase that by using the pre-processed expression values no informative similarities or correlations between exposures can be identified.

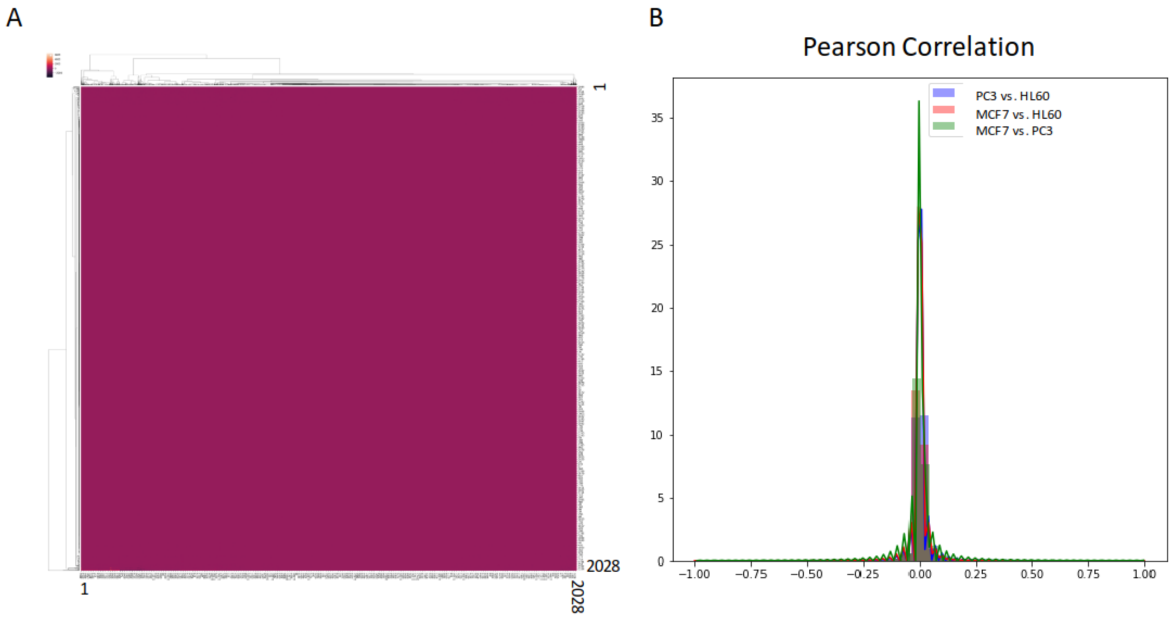

Figure 2: A) Heatmap of all 676 chemicals in all three cell lines based on the expression values of all 11 868 genes. B) Pearson Correlation between all pairs of exposures between two cell lines, showing no correlation.

Comparison of the CMap Biological Systems

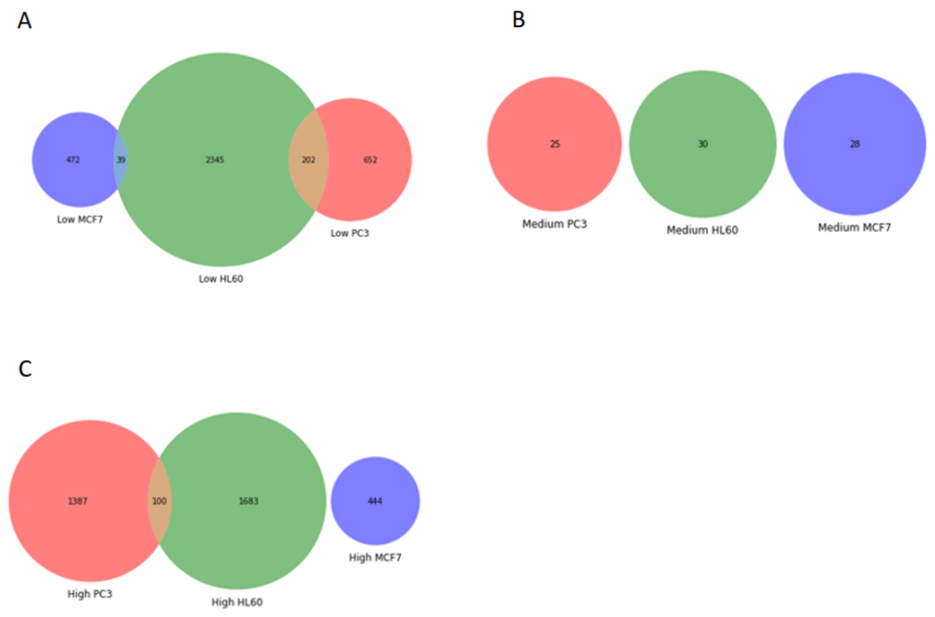

Figure 3: Venn Diagram of the assigned expression classes for each biological system's control gene expression values. The low overlap indicates that the overall gene expression profiles are highly different for the systems. A) low, B) Medium & C) High expressed genes. Blue: MCF7, Green: HL60 & Red: PC3.

Convergence of the feature vector

For each CMap instance a feature vector was computed, as described in the main text, based on the top 10, 200, 500, 2000, 5000 & 10000 most differential genes. The Cosine Distance and Pearson Correlation were estimated on each biological system between all pairwise exposures. The mean and standard deviation of these distances/ correlations are plotted in figure 4.

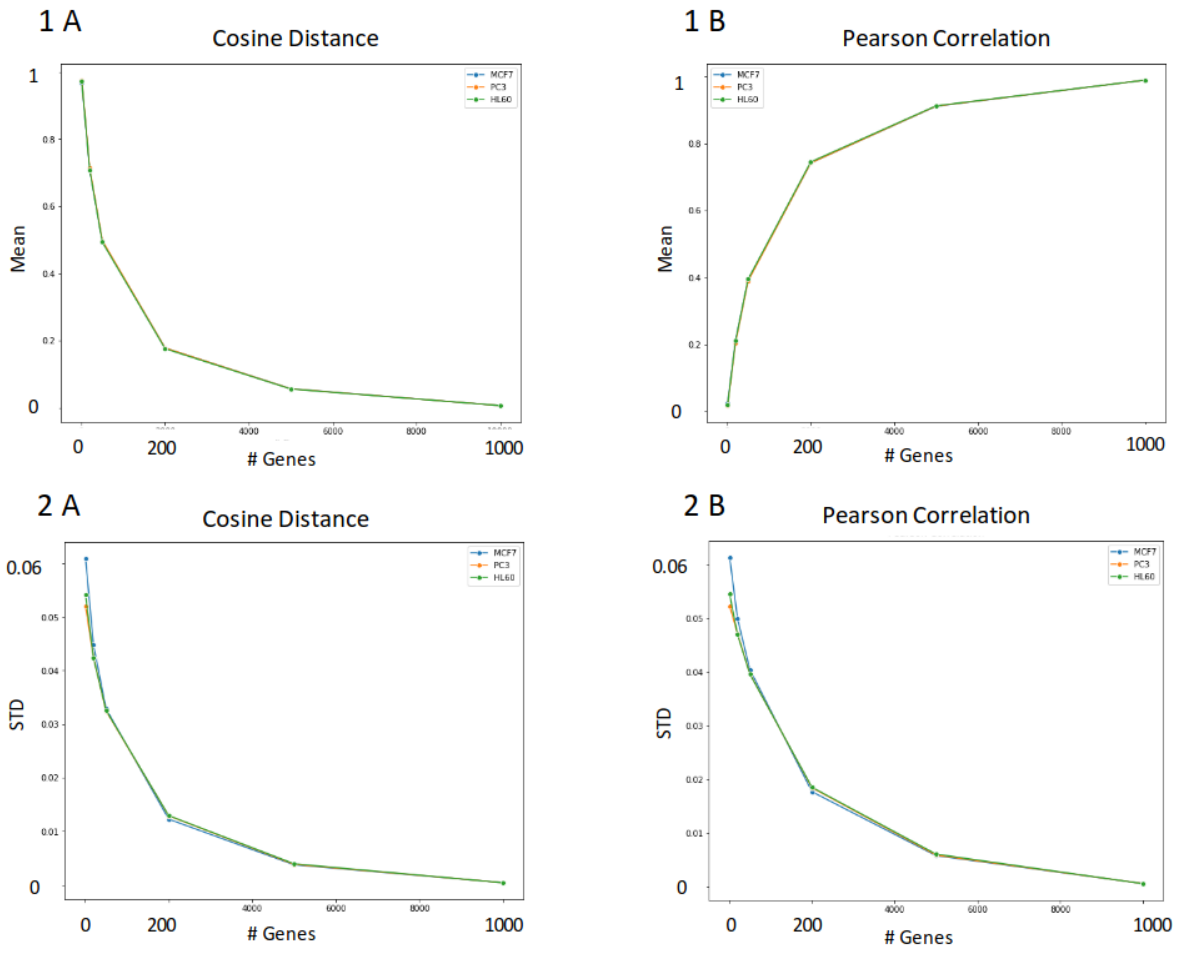

Figure 4: Row 1) The mean distance / correlation between each pair of exposures for all three biological systems for the 10, 200, 500, 2000, 5000 & 10000 most differential genes, showing the convergence to 0/ 1 for larger gene sets and 1/ 0 for small gene sets. Row 2) The standard deviation of the distance / correlation between each pair of exposures for all three biological systems for the 10, 200, 500, 2000, 5000 & 10000 most differential genes, showing a drop in diversity with the increase of the gene set size. Blue: MCF7, Red: PC3 & Green: HL60

Comparison to traditional Methods

Fraction ATC Level 3

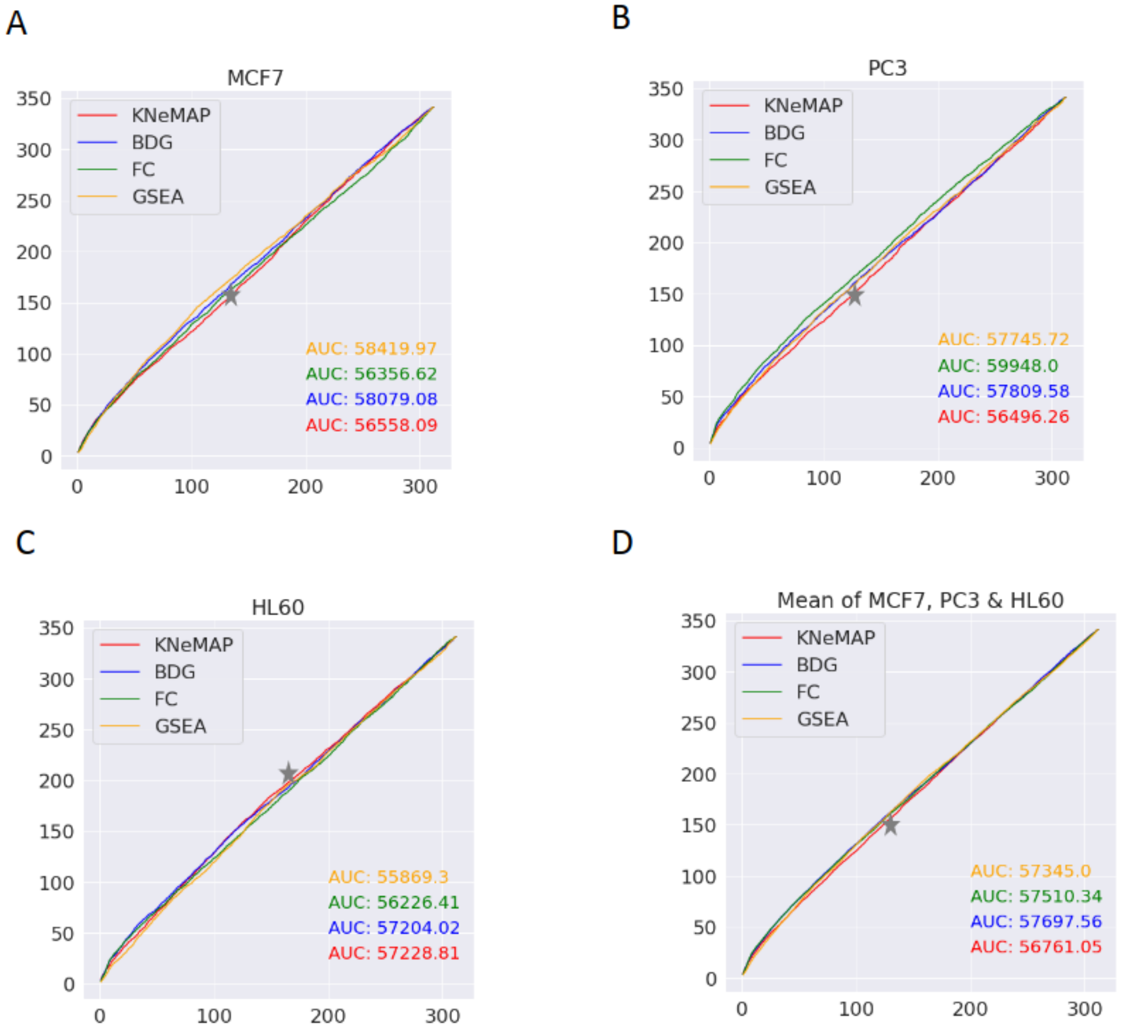

Figure 5: Performance Comparison of KNeMAP, BDG, GSEA and FC to identify similar compounds based on ATC codes (level 3) in the same data set. For MCF7 (A), PC3 (B), HL60 (C) and the average on all three (D). The performance is highly similar between the methods, as indicated by their similar AUC score, of which a high AUC score indicates the best performance. Blue: top 200 deregulated genes (BDG), Red: KNeMAP, Orange: GSEA & Green: All $logFC * -log(Pval)$ Values (FC); the stars are indicators of the KNeMAP line, used to improve inclusivity of the figure.

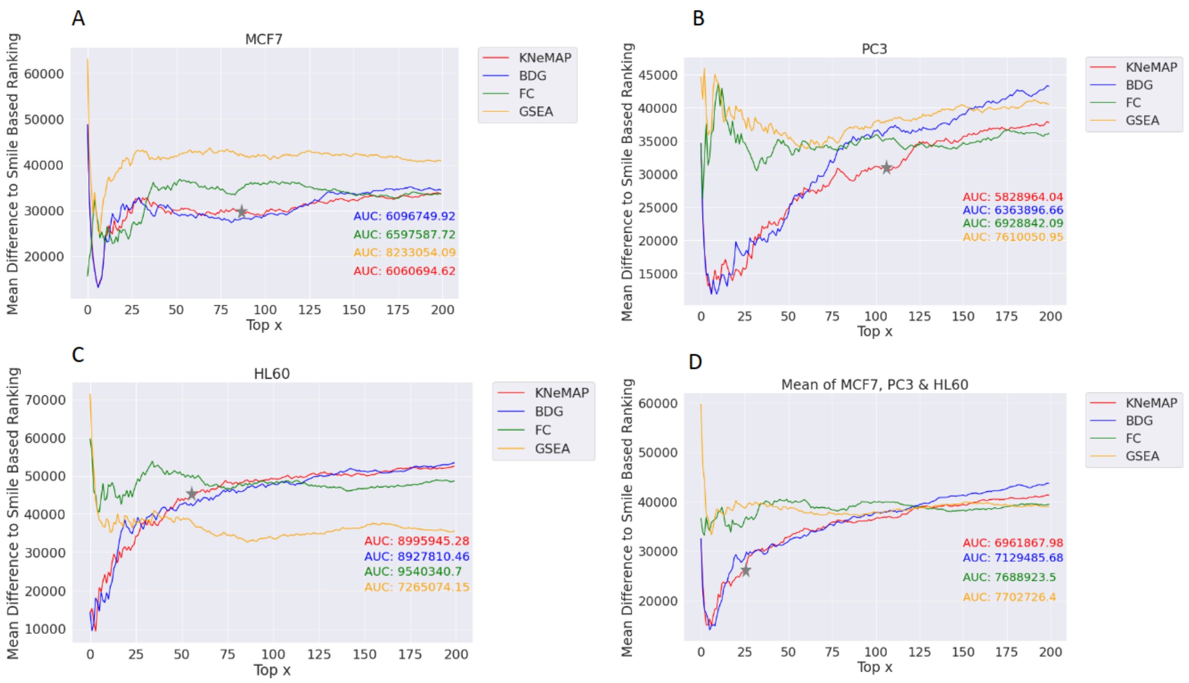

Figure 6: Comparison of the four different methodologies, showcasing how similar their pairwise compound ranks are to a SMILE based ranking for the top 200 pairs. The y-axis values contain the average difference between a compound ranking to the SMILE based compound ranking for a method's top x pairs, which is indicated by the x-axis values. The performance is displayed for all three biological systems (A-C) and D showcases the average performance over all three biological systems. While differences in performance can be observed, on average our method outperforms a gene based and expression value based method (red), where lower y-values indicate a higher agreement with the SMILE based ranking, which is showcased by the lowest area under the curve (AUC). Distances between SMILEs are computed based on the Levenshtein distance and the distance between compounds for the four compared methods are based on a cosine distance. Blue: top 200 deregulated genes (BDG), Red: KNeMAP, Orange: GSEA & Green: All $logFC * -log(Pval)$ Values (FC); the stars are indicators of the KNeMAP line, used to improve inclusivity of the figure.

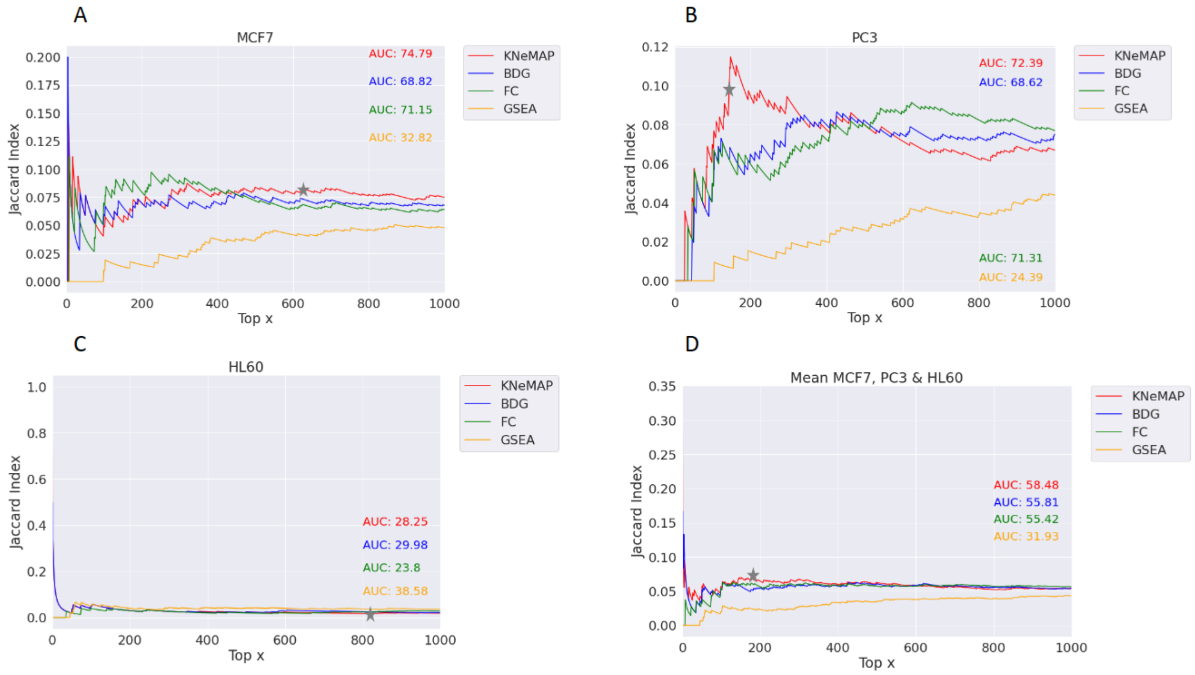

Figure 7: Comparison of the four different methodologies, showcasing how similar their pairwise compound ranks are to a SMILE based ranking for the top 1000 pairs. For each method the top x pairs are selected and the jaccard index is estimated against the top x pairs based on a SMILE based ranking. The performance is displayed for all three biological systems (A-C) and D showcases the average performance over all three biological systems. While differences in performance can be observed, on average our method outperforms a gene based and expression value based method (red), where higher y-values indicate a higher agreement with the SMILE based ranking, which is showcased by the highest area under the curve (AUC). Distances between SMILEs are computed based on the Levenshtein distance and the distance between compounds for the four compared methods are based on a cosine distance. Blue: top 200 deregulated genes (BDG), Red: KNeMAP, Orange: GSEA & Green: All $logFC * -log(Pval)$ Values (FC); the stars are indicators of the KNeMAP line, used to improve inclusivity of the figure.

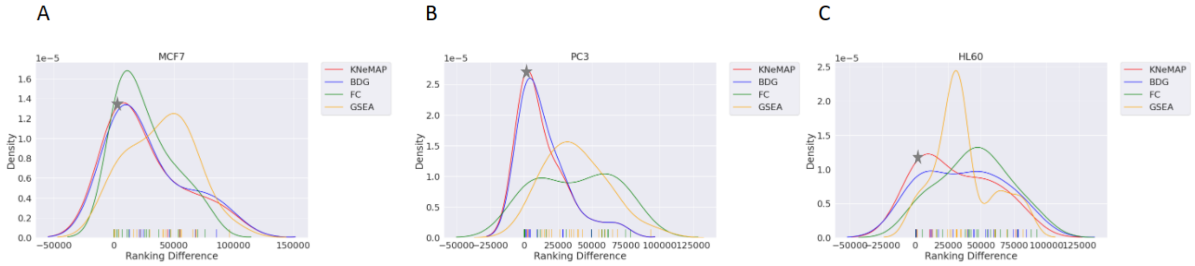

Figure 8: Depicts the density plot of the rank difference between compound pairs (distances between compound pairs have been calculated with a cosine distance) based on KNeMAP, a binary gene based method, a GSEA based method and an expression based method in comparison to a SMILE based ranking (based on the Levenshtein distance). The rank difference for a method's top 20 pairs are displayed, showcasing a general shift to the left for KNeMAP (red), indicating higher agreement with a structural (SMILE) based ranking than for

traditional methods. Blue: top 200 deregulated genes (BDG), Red: KNeMAP, Orange: GSEA & Green: All $logFC * -log(Pval)$ Values (FC); the stars are indicators of the KNeMAP line, used to improve inclusivity of the figure.

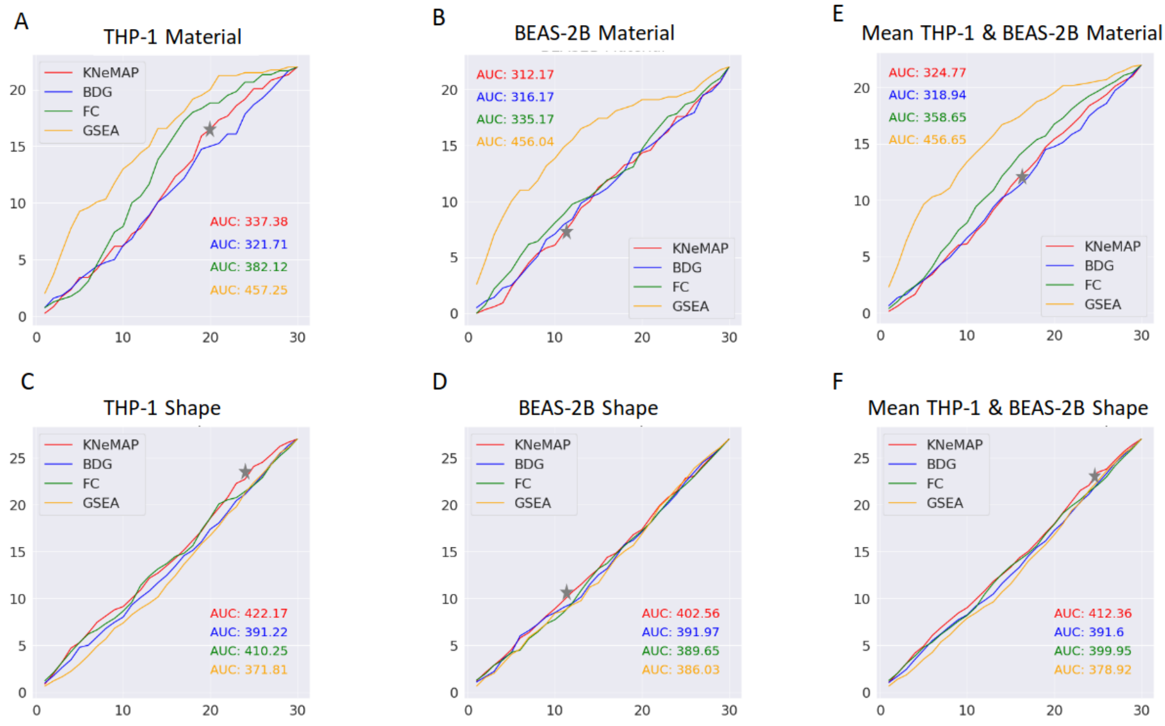

Figure 9: Performance comparison of KNeMAP, BDG, GSEA and FC to identify the same core material (A, B, E) or the same material shape (C, D, F) on the same biological system. On average GSEA and FC are better in identifying the same core material, while KNeMAP outperformed the other methods on average when identifying structural similarities (F). Blue: top 200 deregulated genes (BDG), Red: KNeMAP, Orange: GSEA & Green: All $logFC * -log(Pval)$ Values (FC); the stars are indicators of the KNeMAP line, used to improve inclusivity of the figure.

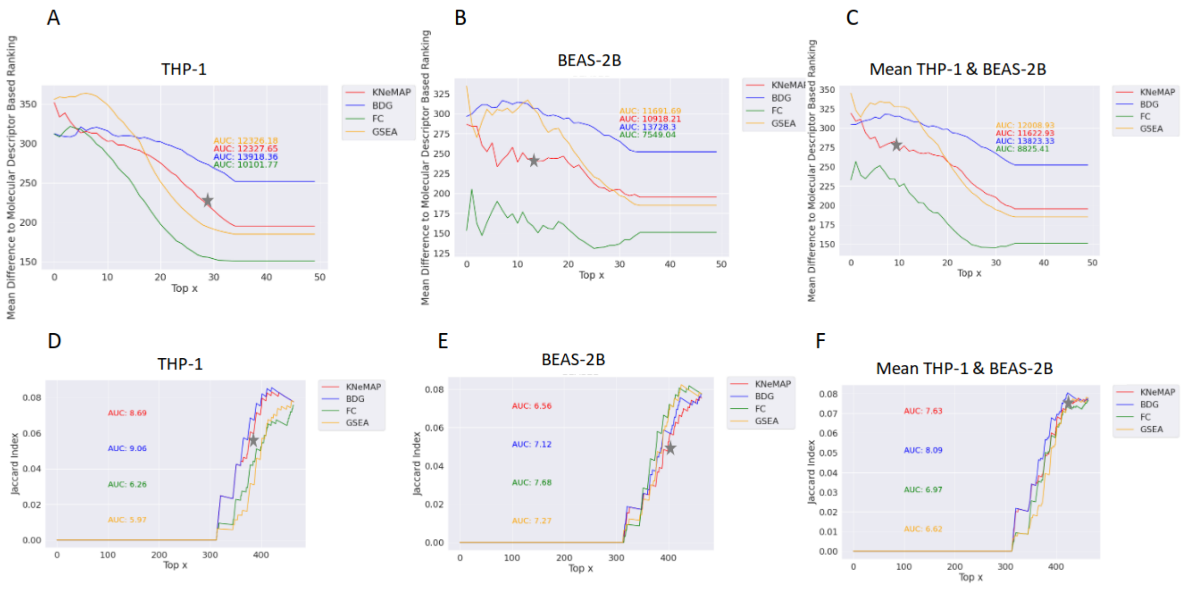

Figure 10: Comparison of the four different methodologies, showcasing how similar their pairwise compound ranks are to a molecular descriptor based ranking for the top 50 (A, B, C) and all (D, E, F) pairs. For each method the top x pairs are selected and the average difference between a compound ranking to the molecular descriptor based compound ranking are estimated (A, B, C) as well as a jaccard index is estimated against the top x pairs against the molecular descriptor based ranking (D, E, F). The performance is displayed for both biological systems (A, B, D, E) and C & F showcase the average performance over both biological systems. While KNeMAP does not show the best performance on average, it is for both metrics the second best performing one, while the other three measures show differences in their performance. FC shows the best performance in C, as indicated by its lowest AUC score, while BDG shows the strongest performance on F as indicated by its highest AUC score. Though it is to note that on the jaccard index based metric, all methods show very little agreement with the molecular descriptor based ranking. Distances between ENMs are computed based on the cosine distance between their molecular descriptors and the distance between compounds for the four compared methods are based on a cosine distance. Blue: top 200 deregulated genes (BDG), Red: KNeMAP, Orange: GSEA & Green: All $logFC * -log(Pval)$ Values (FC); the stars are indicators of the KNeMAP line, used to improve inclusivity of the figure.

Distribution of Similarities Across the Datasets

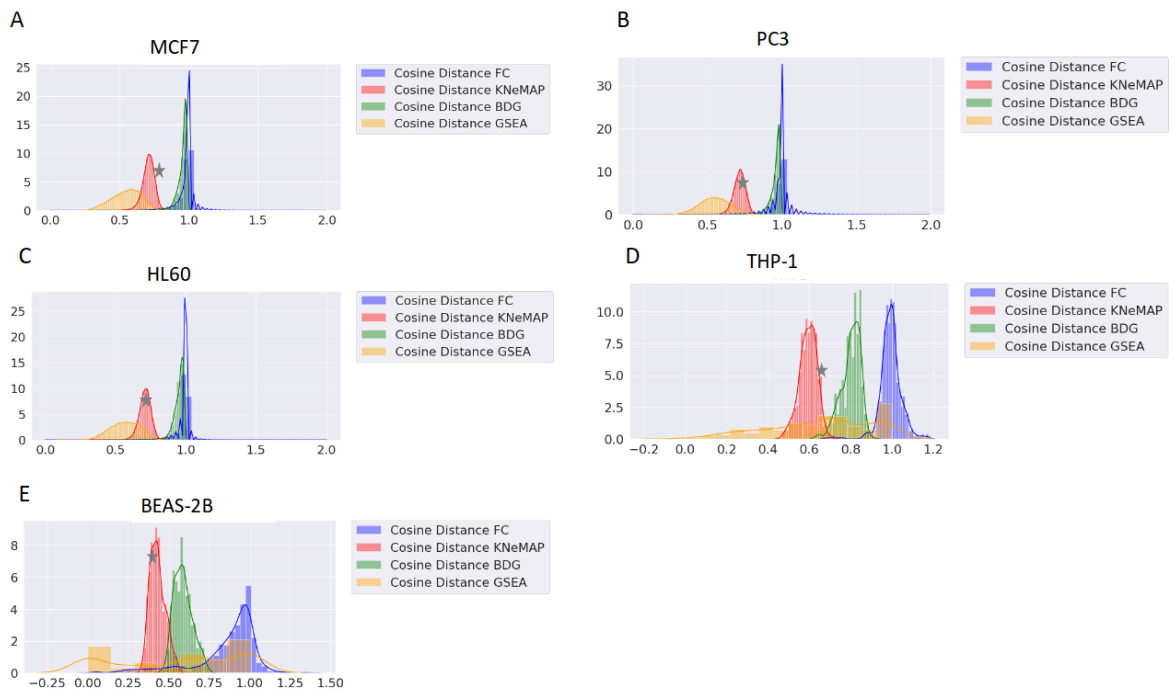

Figure 11: Cosine Distance between all pairwise exposures on the MCF7 (A), PC3 (B), HL60 (C), THP-1 (D) and BEAS-2B (E) data set, comparing the value distributions of the described binary and mapped feature vector. Blue: top 200 deregulated genes (BDG), Red: KNeMAP, Orange: GSEA & Green: All $logFC * -log(Pval)$ Values (FC); the stars are indicators of the KNeMAP line, used to improve inclusivity of the figure.

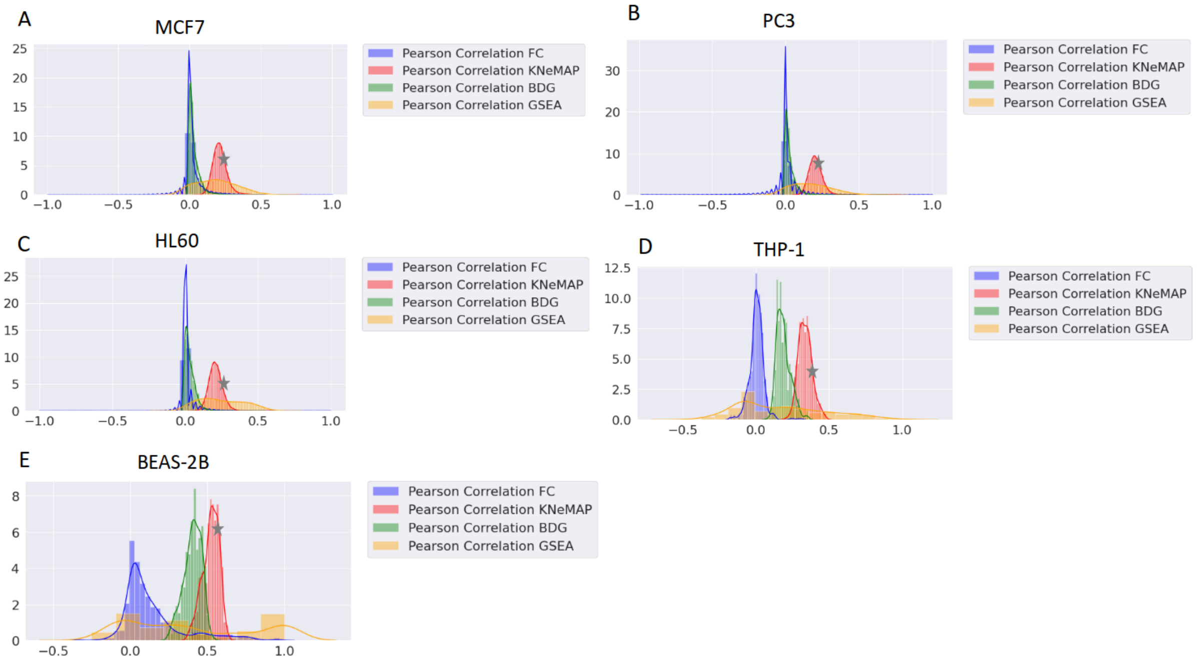

Figure 12: Pearson Correlation between all pairwise exposures on the MCF7 (A), PC3 (B), HL60 (C), THP-1 (D) and BEAS-2B (E) data set, comparing the value distributions of the described binary and mapped feature vector. Blue: top 200 deregulated genes (BDG), Red: KNeMAP, Orange: GSEA & Green: All *logFC* * −*log*(*Pval*) Values (FC); the stars are indicators of the KNeMAP line, used to improve inclusivity of the figure.

Impact of Noise Corrupted Data

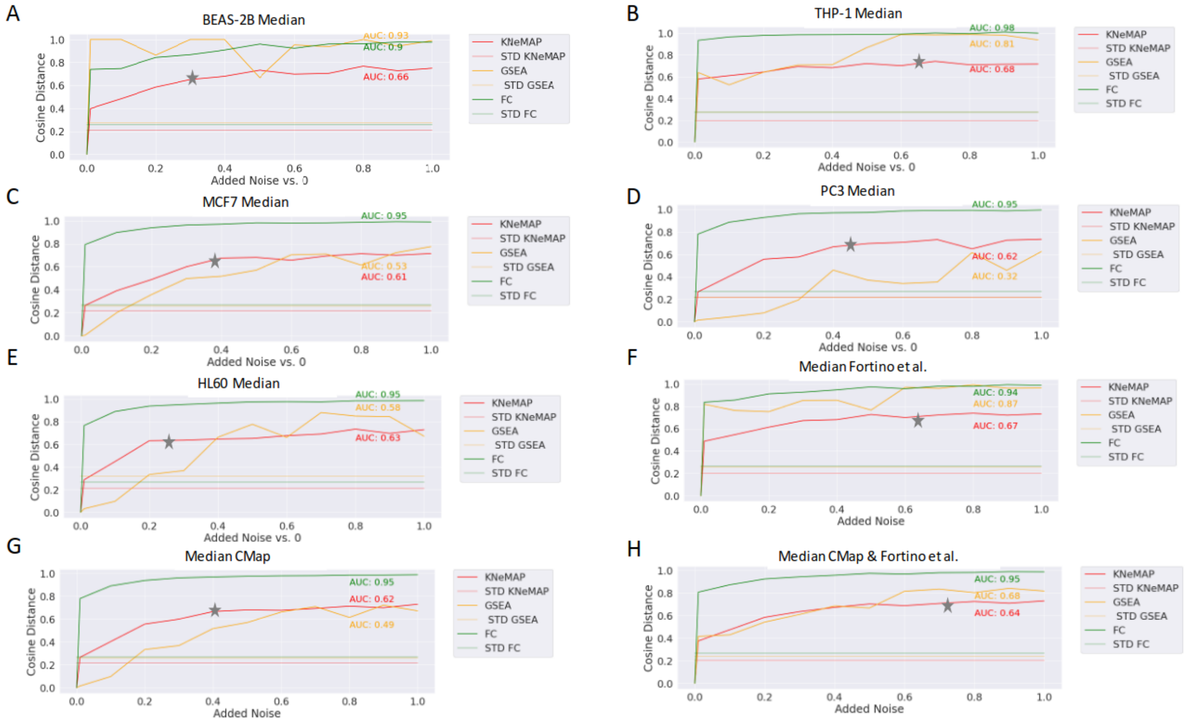

Figure 13: Median cosine distance between KNeMAP (red), GSEA (orange) and FC (green) based vectors with increased levels of added noise to the gene expression values across all five biological systems (A-E). The noise levels are on the x-axis, the mean cosine distance

on the y-axis. KNeMAP vs. FC vs. GSEA. F) The median performance of the Fortino et al. biological systems. G) The median performance of the CMap biological systems. H) The performance across both datasets, to not bias the results due to the difference in number of available biological systems, the median of the media for each dataset was calculated. The stars are indicators of the KNeMAP line, used to improve inclusivity of the figure.

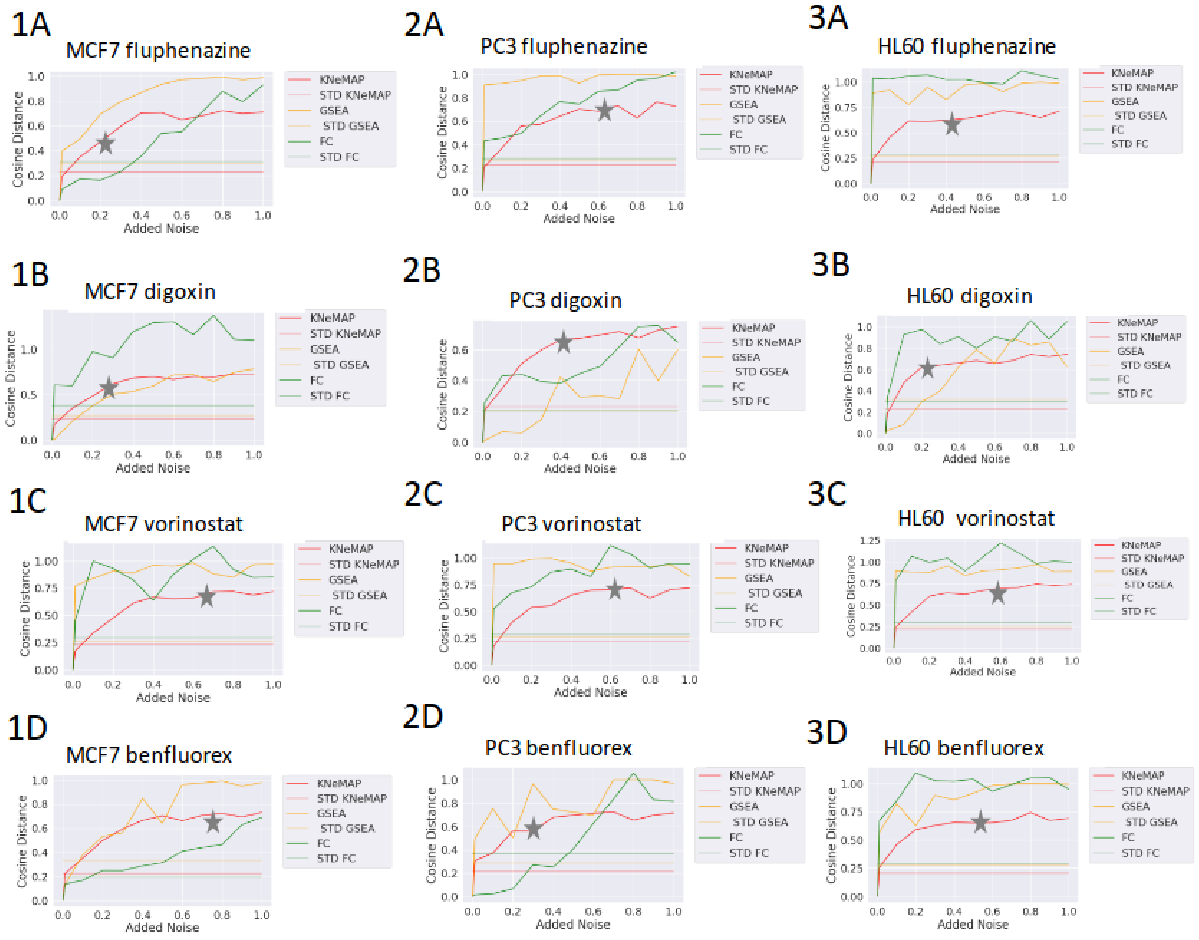

Figure 14: Selected examples of compounds and their cosine distance with increasing levels of added noise to their baseline values. KNeMAP (red) is compared to the FC (green) & GSEA (orange) method. A) depicts the performance of fluphenazine, a phenothiazine in the set of identified 38 CMap compounds (1-3). B) shows the digoxin, a cardiac glycoside in our set of identified 38 CMap compounds (1-3) and C) & D) show two other compounds not in the identified set. The stars are indicators of the KNeMAP line, used to improve inclusivity of the figure.

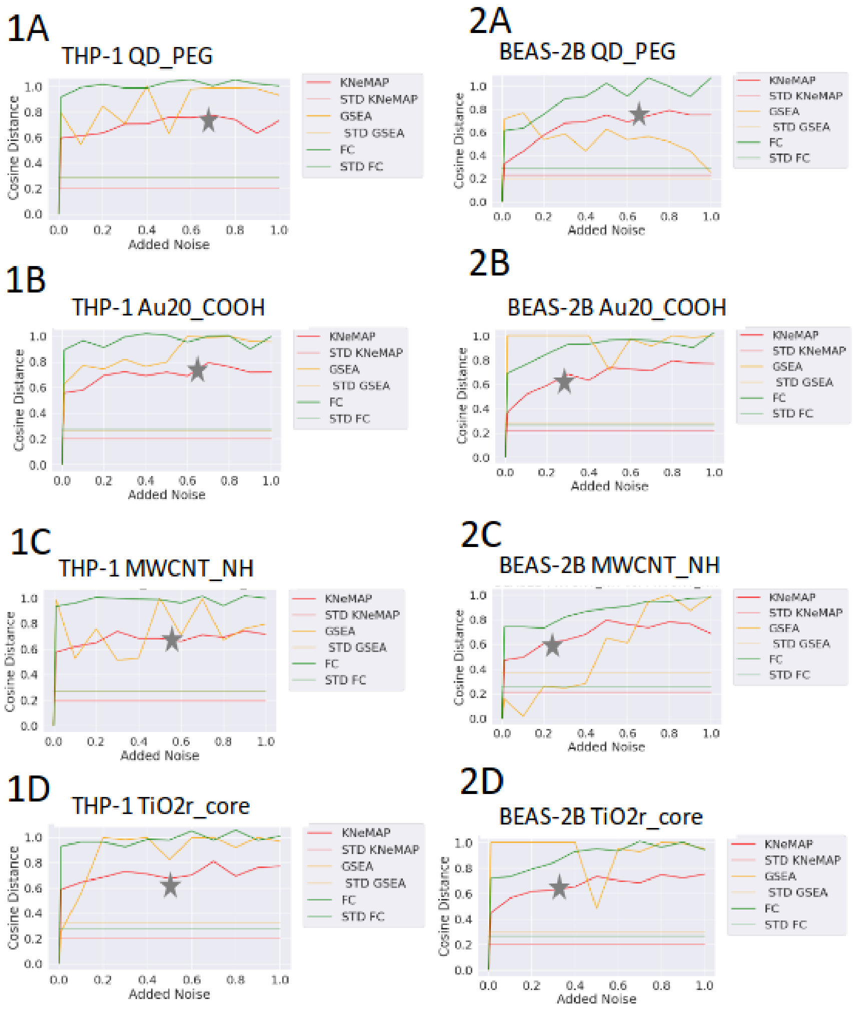

Figure 15: Selected examples of compounds and their cosine distance with increasing levels of added noise to their baseline values. KNeMAP (red) is compared to the FC (green) & GSEA (orange) method. A) depicts the performance of QD_PEG, an ENM in the identified set for the Fortino et al. data (1-2) . B) shows Au20_COOH, a ENM in the identified set for the Fortino et al. data (1-2) and C) & D) show two other compounds not in the identified set. The stars are indicators of the KNeMAP line, used to improve inclusivity of the figure.

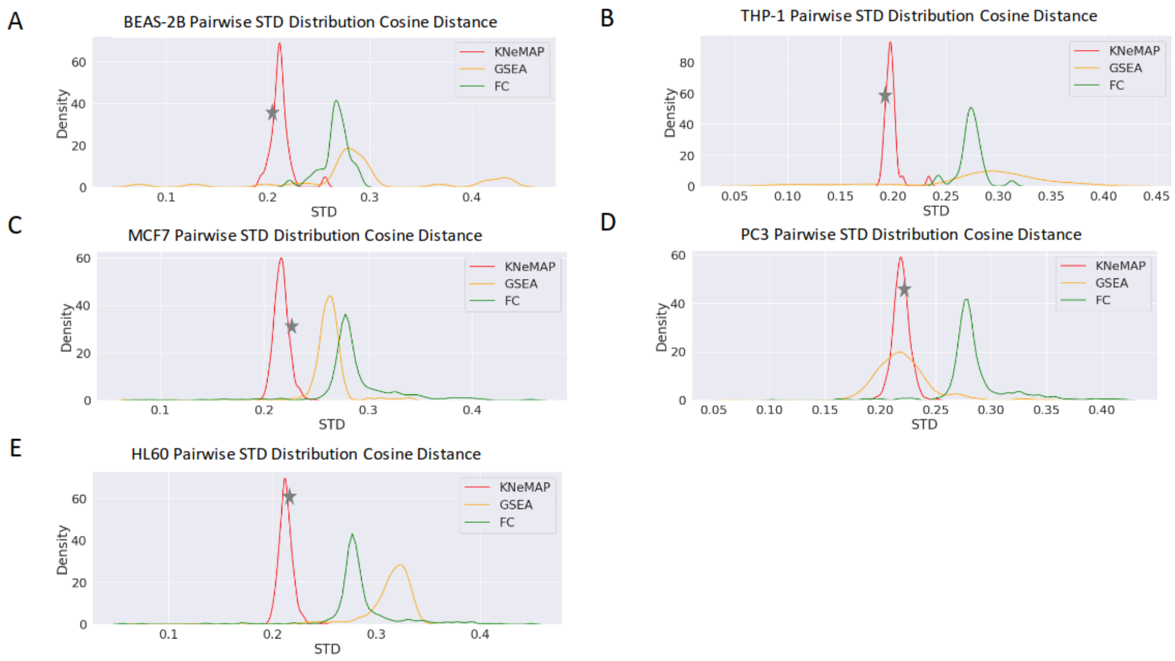

Figure 16: Standard deviation of the cosine distance between the original data and noisy data across all 5 biological systems.KNeMAP (red) is compared to the FC (green) & GSEA (orange) method. The stars are indicators of the KNeMAP line, used to improve inclusivity of the figure.

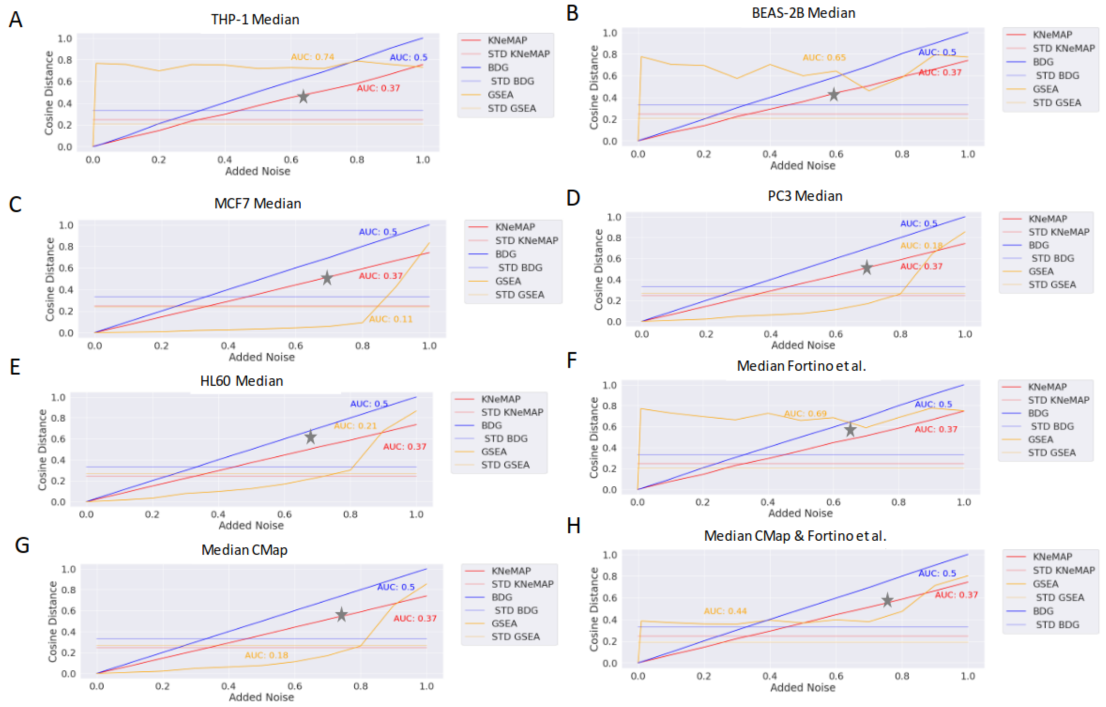

Figure 17: Median cosine distance between KNeMAP (red), GSEA (orange) and BDG (blue) based vectors with increased levels of added noise to the selected 200 most deregulated genes across all five biological systems (A-E). The noise levels are on the x-axis, the mean cosine distance on the y-axis. KNeMAP vs. FC vs. GSEA. F) The median performance of the Fortino et al. biological systems. G) The median performance of the CMap biological

systems. H) The performance across both datasets, to not bias the results due to the difference in number of available biological systems, the median of the media for each dataset was calculated. The stars are indicators of the KNeMAP line, used to improve inclusivity of the figure.

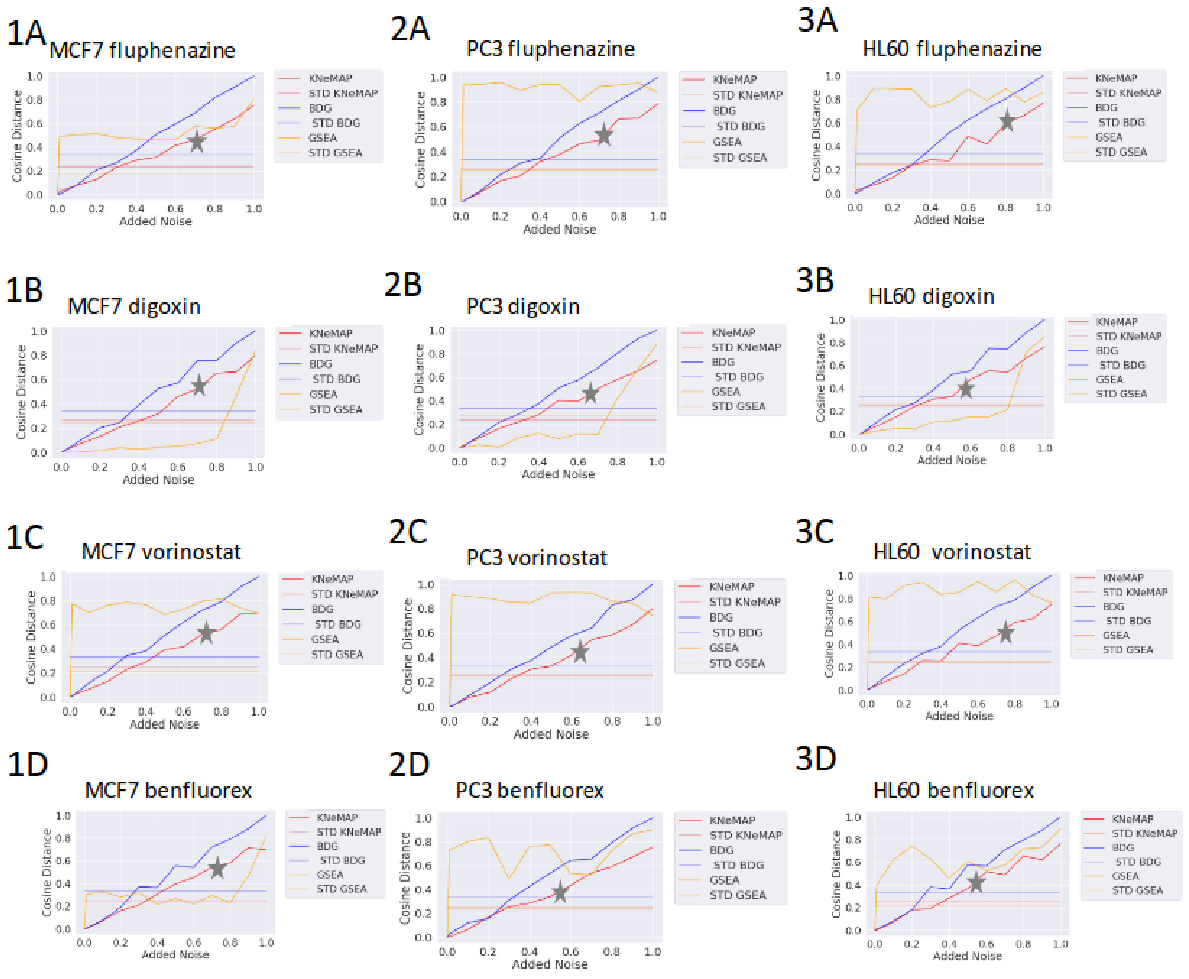

Figure 18: Selected examples of compounds and their cosine distance with increasing levels of added noise to the selected top 200 most deregulated genes. KNeMAP (red) is compared to the BDG (blue) and GSEA (orange) method. A) depicts the performance of fluphenazine, a phenothiazine in the set of identified 38 compounds. B) shows the digoxin, a cardiac glycoside in our set of identified 38 compounds and C) & D) show two other compounds not in the set of 38 CMap compounds. The stars are indicators of the KNeMAP line, used to improve inclusivity of the figure.

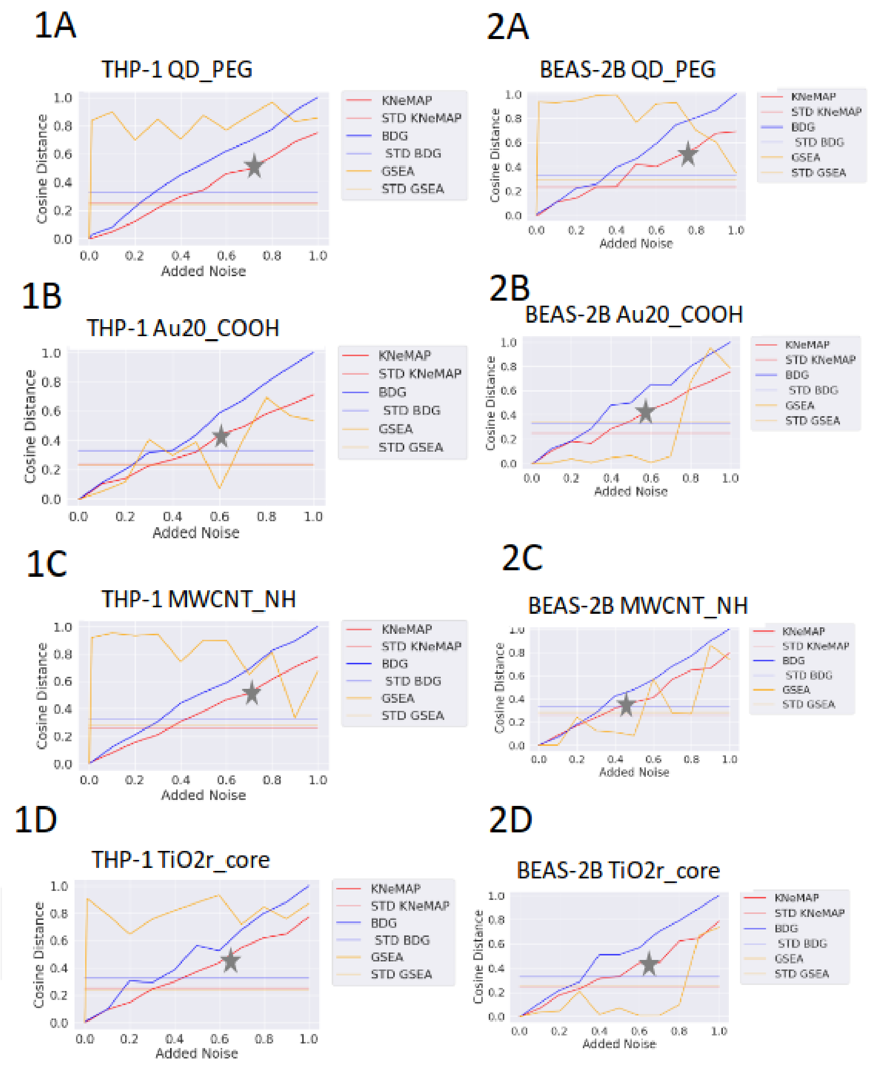

Figure 19: Selected examples of compounds and their cosine distance with increasing levels of added noise to the selected top 200 most deregulated genes. KNeMAP (red) is compared to the BDG (blue) & GSEA (orange) method. A) depicts the performance of QD_PEG, an ENM in the identified set for the Fortino et al. data (1-2) . B) shows Au20_COOH, a ENM in the identified set for the Fortino et al. data (1-2) and C) & D) show two other compounds not in the identified set. The stars are indicators of the KNeMAP line, used to improve inclusivity of the figure.

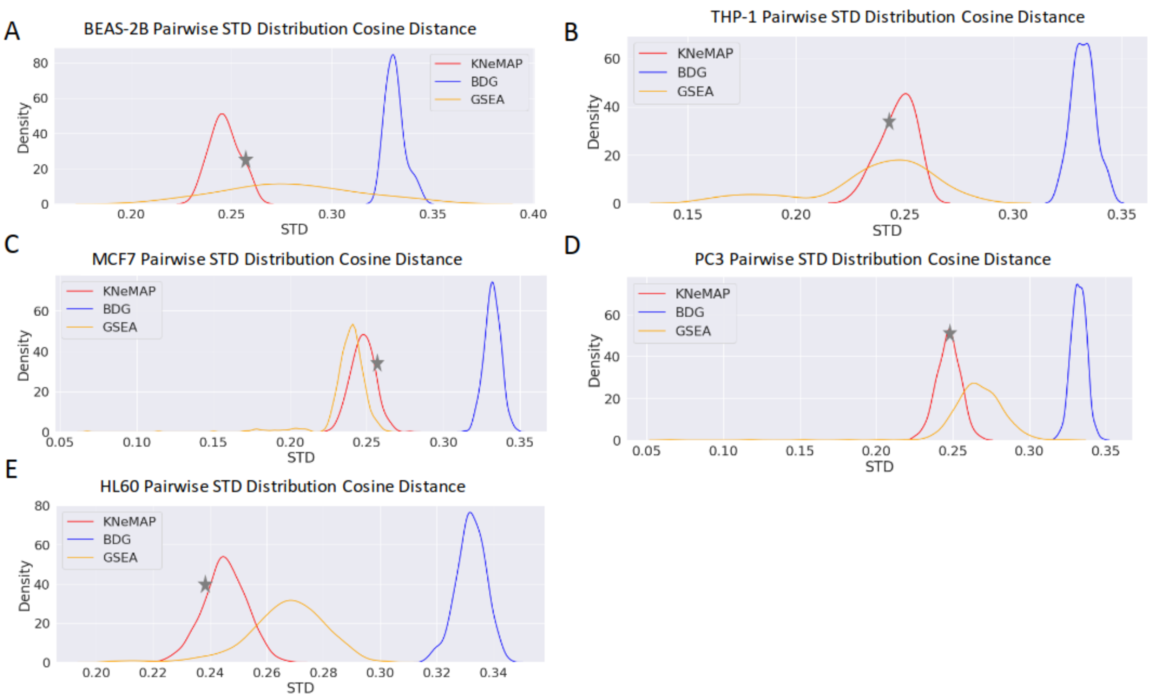

Figure 20: Standard deviation of the cosine distance between the original data and noisy data (perturbation of the top 200 most deregulated genes) across all 5 biological systems. KNeMAP (red) is compared to the BDG (blue) & GSEA (orange) method. The stars are indicators of the KNeMAP line, used to improve inclusivity of the figure.

Stability of KNeMAP Vector Across Different Biological Systems

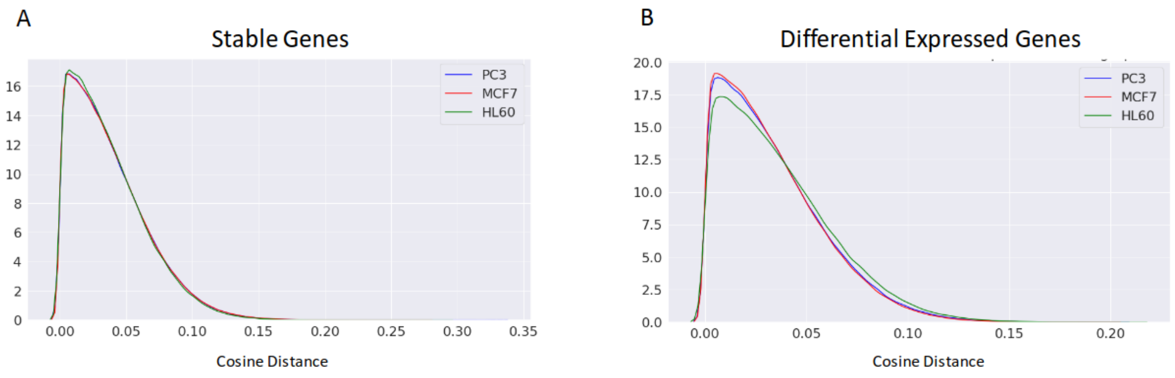

Figure 21: Distribution of cosine distance between KNeMAP fingerprint vectors computed on all available FCP data points or only on genes that are stable across all three biological systems (A) as well as only based on genes that are differential expressed between the steady state CMap cell lines (B). A gene is differential expressed, if it is differential expressed in at least one cell line pair. Both metrics show that the majority of KNeMAP vectors are very little affected by the differences in the biological systems.

Identification, Description and Analysis of the Identified CMap Drugs

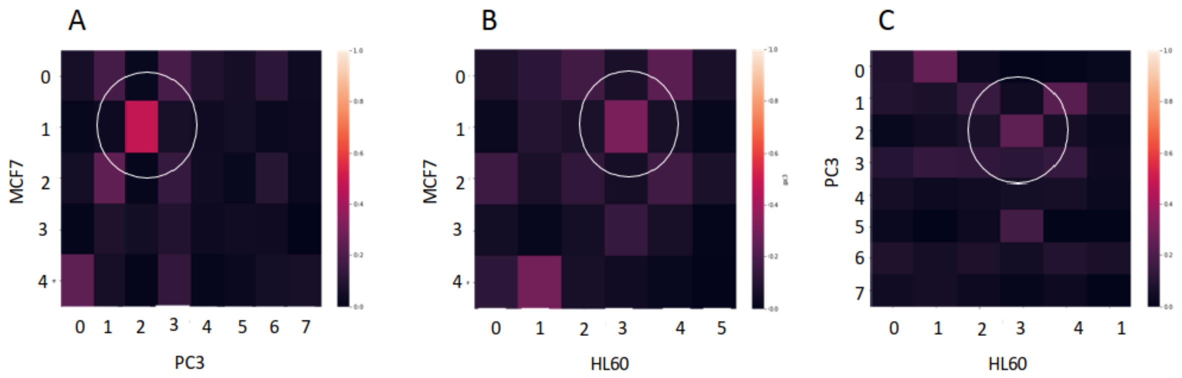

Figure 22: Jaccard Index between cluster pairs of different biological systems. A) MCF7 vs. PC3, B) MCF7 vs. HL60 & C) PC3 vs. HL60

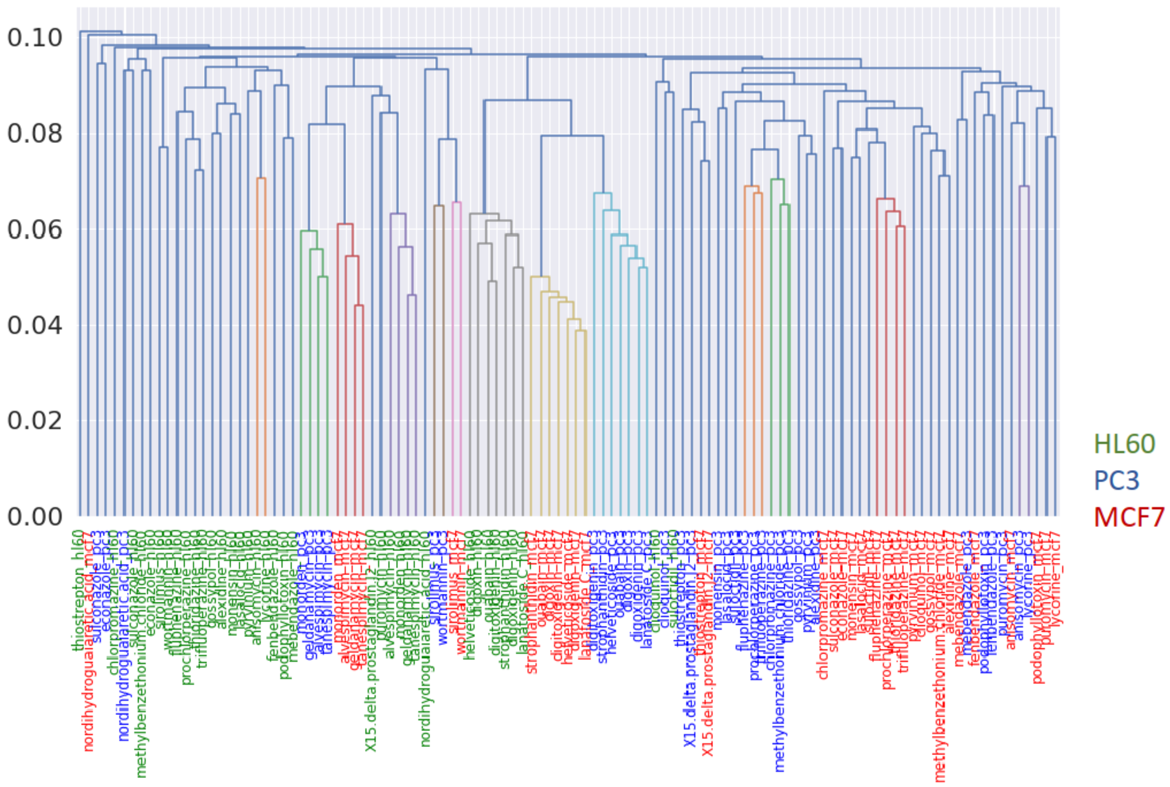

Figure 23: Hierarchical clustering between the identified 38 compounds, colored by their biological system, showcasing a dominance of clustering by biological system instead of compound. This can indicate a difference in MOA on the different biological systems. The linkage has been calculated with Python's scipy (Virtanen *et al.*, 2020b) *scipy.cluster.hierarchy.linkage(metric="euclidean", method="average").*

Comparison of Transcriptomic Profiles Across Different Cell Lines Identifies Drugs with a System Dependent Similar Mechanism of Action

The CMap data contains three cancer cell lines, PC3, MCF7 and HL60. PC3 is an epithelial cell line derived from bone metastasis of a grade IV prostate adenocarcinoma. Similarly, MCF7 are epithelial cells of metastatic breast adenocarcinoma, expressing the oncogene WNT7B and inhibited by the tumor necrosis factor (TNF). HL60 are promyeloblast derived from peripheral blood of a patient with acute promyelocytic leukemia. With respect to the previous two adherent cell lines, HL60 cells grow in suspension, and treatment with specific drugs can induce differentiation.

In order to identify biological system independent compound groups, a jaccard index was estimated between each cluster pair (s. Methods) between the different biological systems, as shown in figure 22, from which one highly similar cluster between the systems could be identified. The highest agreement can be observed between MCF7 and PC3 (supplementary figure 22A) for clustered 1 and 2 (cluster indices start at 0) respectively, sharing 70 compounds in total. When analyzing the cluster with most agreement in each cell line we found 38 compounds in their intersection. This corresponds to MCF7 cluster 1, PC3 cluster 2 and HL60 cluster 3. From now on, we consider these 38 chemicals during further analysis.

Description of the 38 Drugs Reveals a Dominance of Antimicrobial and Phenothiazine Compounds

In order to investigate if there is a functional reason behind why we can observe this similar behavior between the set of 38 compounds independent from the exposed biological system, we described the compounds based on their known therapeutic indication and protein targets. The list of identified 38 compounds is shown in supplementary file 2.

We investigated whether these drugs shared commonalities in their pharmacological indication, as well as in the anatomical district they affect. When categorizing the drugs by their assigned ATC codes, 11% of compounds are of class N (Nervous System) and D (Dermatologicals), however close to 60% of compounds have no ATC class assigned. The most prominent ATC level 2 classes are N05 (Psycholeptics) at 11%, G01 (Gynecological antiinfectives and antiseptics) at 5% and D01 (Antifungals for dermatological use) at 5%, as shown in figure 26.

Since these compounds did not belong to a homogeneous specific therapeutic category, we retrieved their molecular targets from DrugBank (Wishart *et al.*, 2008, 2006). For 13% of compounds no known assigned gene targets could be retrieved (at the time of the analysis). The most prominent PANTHER Protein Families (Thomas *et al.*, 2003; Mi *et al.*, 2021) present in the identified 38 drugs targets belong to the Adrenergic Receptor-Related G-Protein Coupled Receptor group and the 5-Hydroxytryptamine Receptor group, as shown in supplementary figure 25.

Antimicrobial compounds are a very heterogeneous class, containing compounds with various MOAs and targets. Antimicrobial peptides have been often proposed as possible repurposed drugs for cancer treatments, possibly explaining their effect on the cell lines

used in the CMap dataset (Jafari *et al.*, 2022). Most of the antimicrobial drugs highlighted by our approach either inhibit protein synthesis, are metal chelator or inhibit tubulin assembly. In the last two decades, multiple iron binding drugs have entered clinical trials for their anti-tumor activity (Lovejoy *et al.*, 2011). Similarly, many natural compounds, or derivatives, have been extensively investigated for their capability of interacting with tubulin and compromise cell motility (Kingston, 2009).

Cardiac glycosides are steroid like compounds usually prescribed for the treatment of cardiovascular diseases and arrhythmias. The increased heart muscle contraction activity is usually achieved by inhibiting the sodium-potassium pump. Since cancer cell lines express specific isoforms of the Na^+/K^+ pump, cardiac glycosides such as digoxin and digitoxin share a proven anticancer effect (Kepp *et al.*, 2012). Similarly, suloctidil shares an ion channel activity.

The heat shock protein (HSP) inhibitors in this cluster are all antineoplastic benzoquinones. Quinones are derivatives of natural compounds which have been successfully used to treat multiple neoplasms. Their chemistry provides important antioxidant, antimicrobial and anti-inflammatory activities (Majolo *et al.*, 2019; Ko *et al.*, 2017).

Finally, four first-generation antipsychotic drugs (trifluoperazine, fluphenazine, chlorpromazine, and thioridazine) share a similar MOA in each of the three cancer cell lines. These compounds are phenothiazines, which target the dopamine D2 receptor (Wu *et al.*, 2016). The observation of a lower incidence of cancer among patients with disorders such as schizophrenia and Parkinson's disease, resulted in testing of D2 receptor inhibitor in tumor cell cultures and animal models, and eventually to drug repurposing. Indeed, it has been proved that cancer cell lines upregulate the dopamine D2 receptor, and that it ties to the stemness level of the tumor (Weissenrieder *et al.*, 2019).

Most of the drugs present in this cluster contain steroid-like, natural compounds. Polycyclic drugs are deeply studied carcinogenic agents, and their chemistry allows interaction with macromolecules and membranes, and altering the oxidative status of the cell, possibly suggesting a shared mechanism of action in each biological system (Becker and Banik, 2014).

Although not all the compounds share a similar target or have a common MOA, all the classes of compounds present in this set have been proven to have an effect on cancer cell lines, ultimately inducing a cytostatic or cytotoxic effect. Since these drugs interact with membranes, cytoskeletal elements and ultimately affect the redox state of the cell, cancer cell lines may be more susceptible to these mechanisms than normal cells. However it is known that response to drug treatment depends on multiple cellular and physiological factors, as well as to the fact that the drug can simultaneously target multiple proteins in the cell (Unsal-Beyge and Tuncbag, 2022). Molecular heterogeneity across the cell lines most probably causes different signaling alterations in response to the same drug, possibly offering a biological explanation to the observed transcriptomic difference.

Investigation of the Chemical Structures Reveals Scaffolds Related to Antimicrobial, Antipsychotic, Antiemetic and Cardiac Related Functions.

In addition to the functional analysis of the drugs, we also investigated if there are structural characteristics that may underlie the observable behavior.

We compared the scaffolds contained in the selected set of 38 drugs towards the whole set of 676 drugs used in this study and found 13 scaffolds statistically enriched based on a benjamini hochberg (Benjamini and Hochberg, 1995) corrected p-value of 0.05 (supplementary table 1). The 13 scaffolds can be divided into two groups based on their hierarchical relationship (figure 27). The first higher level scaffold (figure 27A) contains a scaffold associated with the class of aromatic heteropolycyclic compounds known as phenothiazines (Ohlow and Moosmann, 2011). These are polycyclic aromatic with a linear tricyclic system that consists of two benzene rings linked by a para-thiazine ring. Derivatives of phenothiazine are highly bioactive and well known as lead structure in medicinal chemistry (Bisson *et al.*, 2007). Several derivatives such as, Chlorpromazine, promethazine and methylene blue had a huge impact in the fields of psychiatry, allergy treatment and antimalarial drugs, respectively. Phenothiazines have gained attention as possible multitargeting agents and currently are one of the core sub-structures in drugs targeting the central nervous system as well as in use as antiinflammatory agents (Key heterocycle cores for designing multitargeting molecules, 2018). Different Phenothiazines have been under investigation for their possible antineoplastic activities (Jones, 1985; Abu-Hashem *et al.*, 2021; Choi *et al.*, 2008) and are considered as promising scaffolds against Tuberculosis (Sellamuthu *et al.*, 2018). In addition it is a tricyclic scaffold, well known in drugs to treat nausea and vomiting (supplementary file 2) and a common substructure contained in drugs targeting the Nervous System/ Psycholeptics (ATC code N05)[1]. The second higher level scaffold (figure 27B) is a common substructure in drugs related to cardiac therapy (ATC code C01)[2], of which some are listed in supplementary table 2. It is a multi-cyclic scaffold, with oxygen containing rings, also called cardiac glycosides, a class of organic compounds used in the treatment and management of cardiac insufficiency, arrhythmias, and heart failure as well as are often used in cell biological studies as an inhibitor of the Na-K-ATPase membrane pump (Patel, 2016; Belz *et al.*, 2001).

[1] https://www.ebi.ac.uk/chembl/g/#substructure_search_results/c1ccc2c(c1)Nc1ccccc1S2

[2] https://www.ebi.ac.uk/chembl/g/#substructure_search_results/O%3DC1C%3DC(C2CCC3C2CCC2C4CCC(OC5CCCCO5)CC4CCC23)CO1

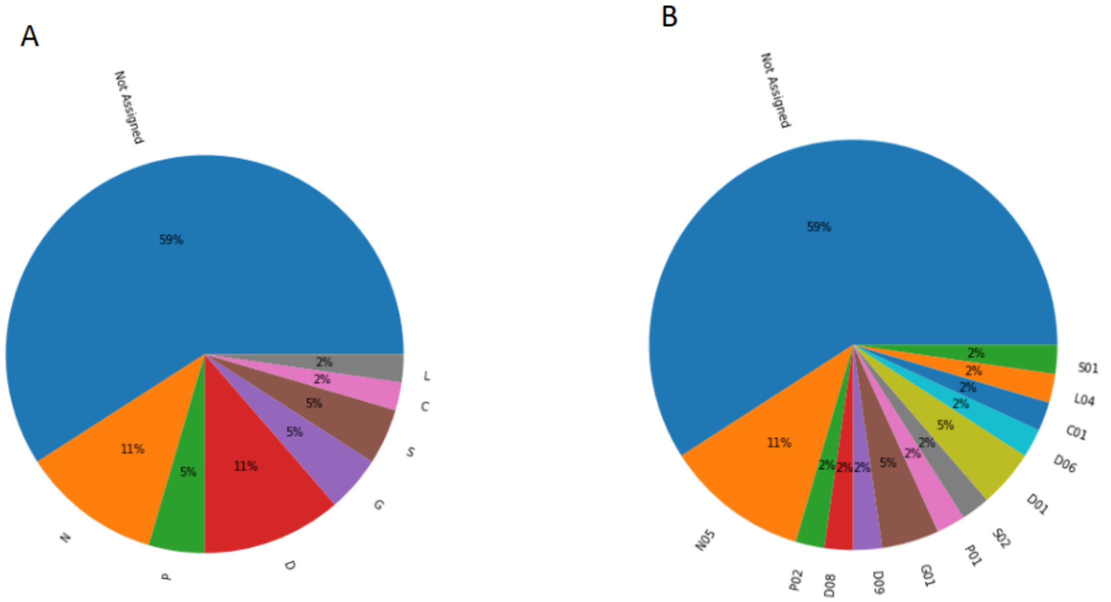

Figure 24: Distribution of Level 1 (A) and Level 2 (B) ATC Codes of the identified 38 drugs. Close to 60% of the identified drugs do not have assigned ATC Codes.

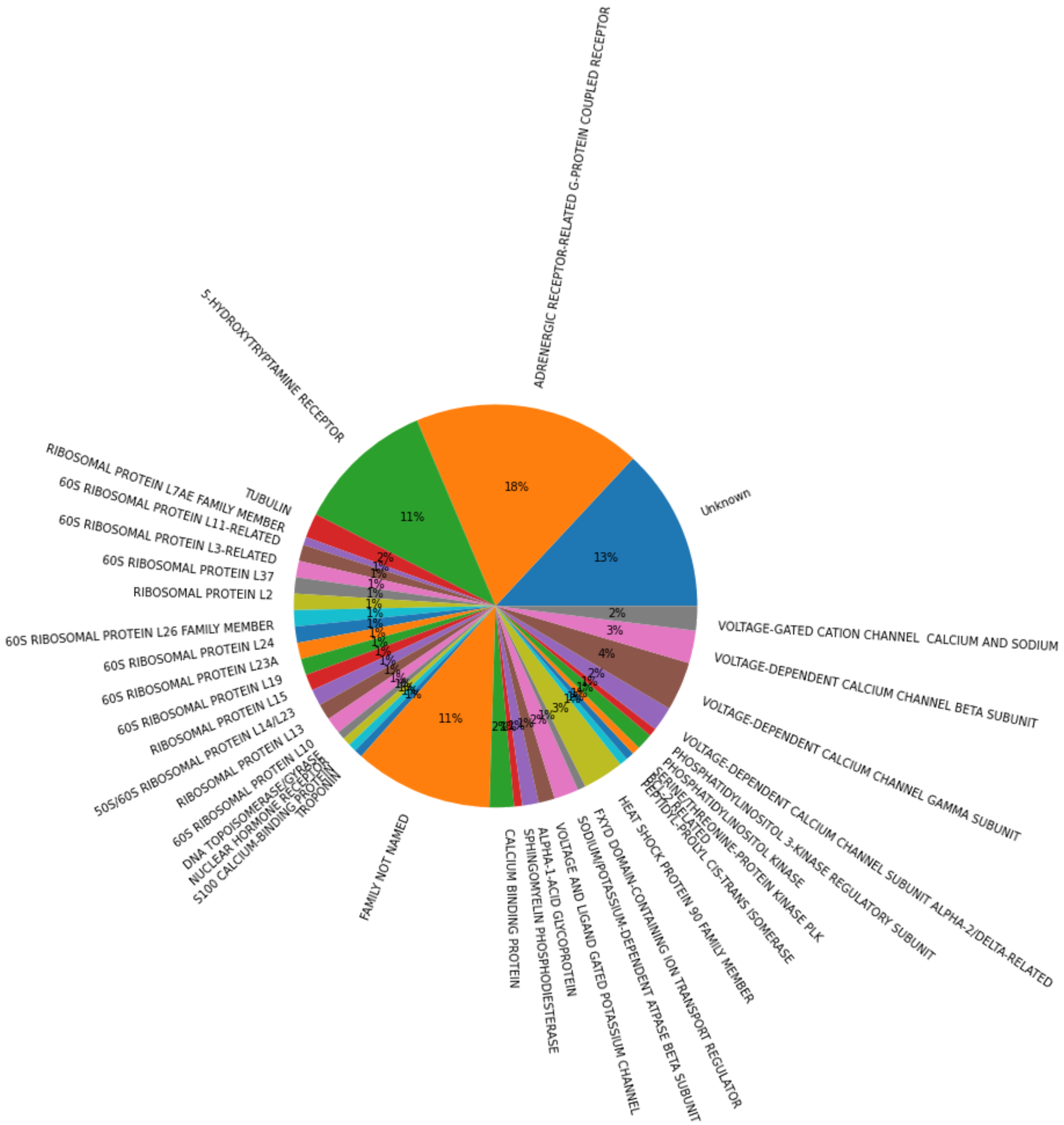

Figure 25: Panther Protein Family Distribution of the Known Drug Targets of the identified 38 drugs.

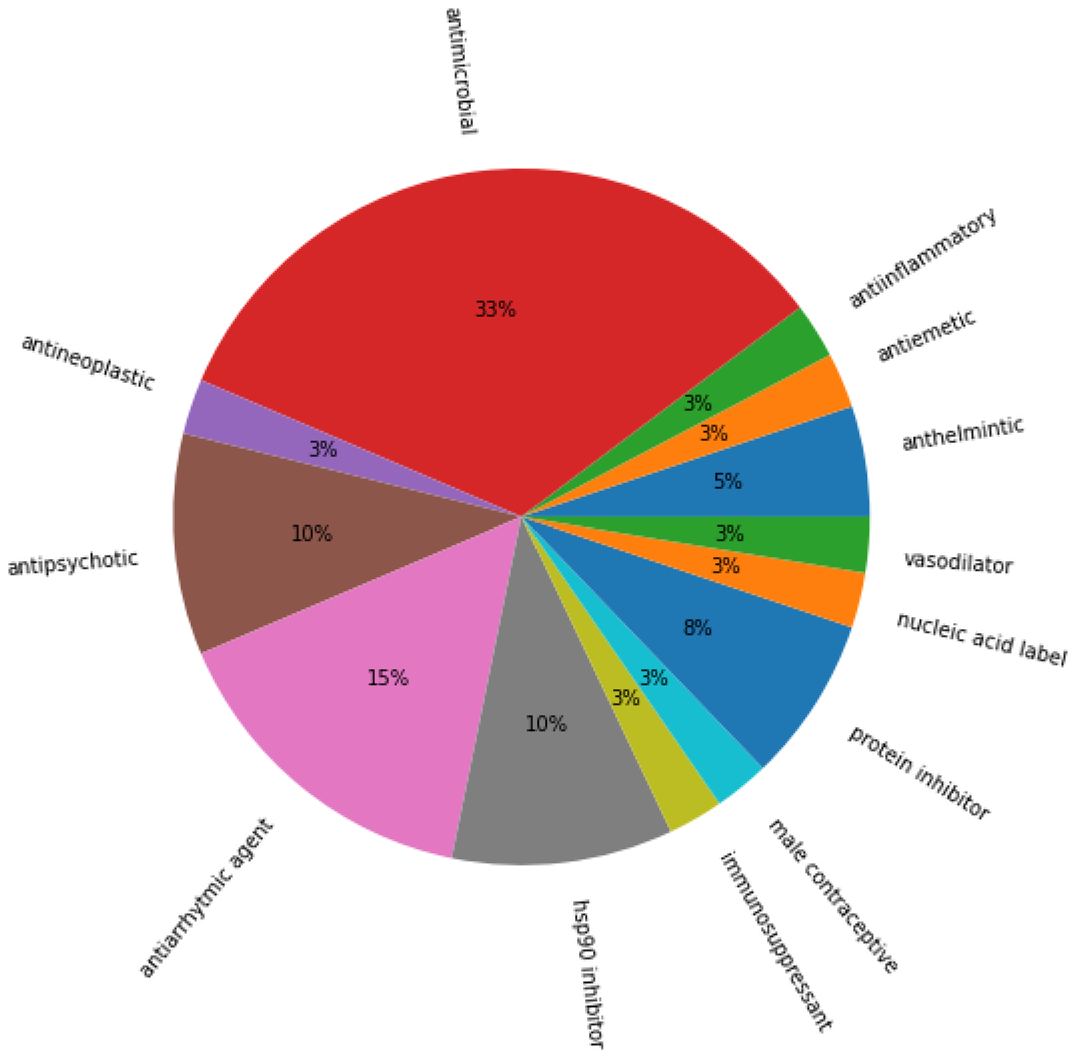

Figure 26: Distribution of Drug Categories of the identified 38 Drugs.

Scaffolds

SMILE		p-value	FDR BH
C1=CSc2ccccc2N1	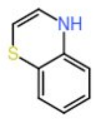	1.13e-04	0.0131

C1=CSC=CN1	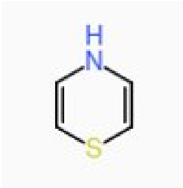	1.13e-04	0.0131
c1ccc2c(c1)Nc1ccccc1S2	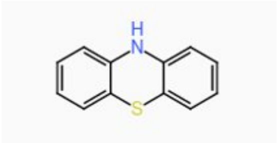	1.13e-04	0.0131
C1CCC2C(C1)CCC1CCCCC12	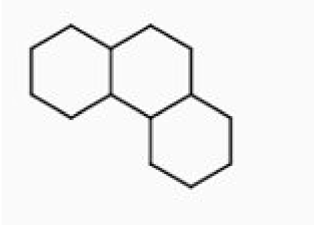	1.07e-05	0.002
O=C1C=CCO1	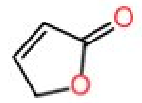	5.9e-05	0.0098
O=C1C=C(C2CCC3C2CCC2C4CCCCC4CCC23)CO1	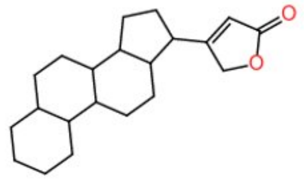	5.55e-07	0.0001
O=C1C=C(C2CCC3C4CCCCC4CCC23)CO1	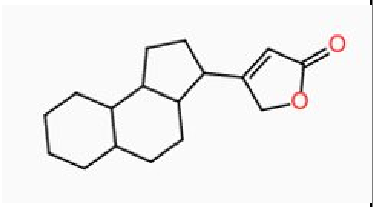	5.55e-07	0.0001
O=C1C=C(C2CCC3CCCCC32)CO1	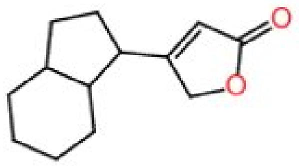	5.55e-07	0.0001
O=C1C=C(C2CCCC2)CO1	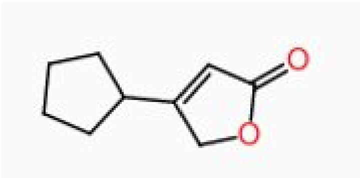	5.55e-07	0.0001

C1CCC2C(C1)CCC1C3CCCC3CCC21	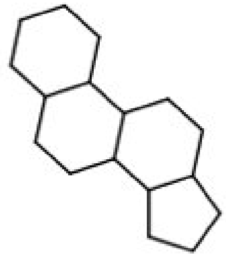	5.55e-07	0.0001
C1CCC(OC2CCC3C(CCC4CCCCC43)C2)OC1	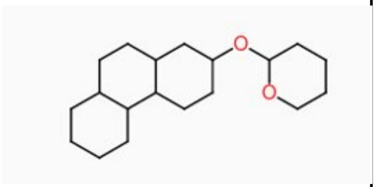	1.98e-04	0.0176
C1CCC(OC2CCC3C(CCC4C5CCCC5CCC34)C2)OC1	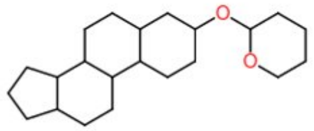	1.98e-04	0.0176
O=C1C=C(C2CCC3C2CCC2C4CCC(OC5CCCCO5)CC4CCC23)CO1	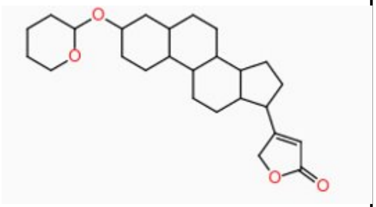	1.98e-04	0.0176

Table 2: Statistically Enriched Scaffolds in the selected set of chemicals and their p-values. The scaffolds have been plotted with: http://www.cheminfo.org/Chemistry/Cheminformatics/Smiles/index.html

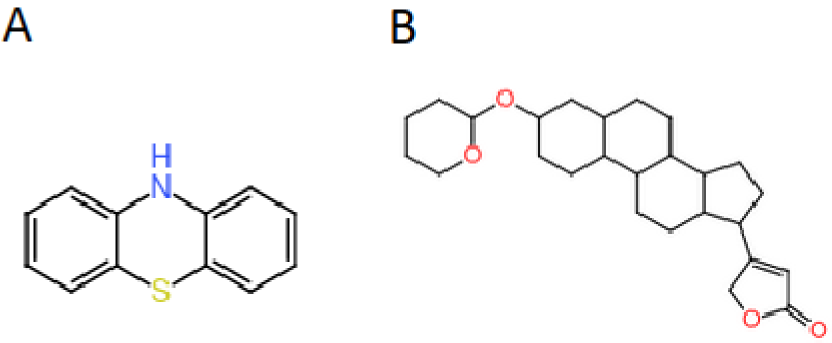

Figure 27: The 2 high level Scaffolds statistically enriched in the identified set of 38 compounds. The scaffolds have been plotted with: http://www.cheminfo.org/Chemistry/Cheminformatics/Smiles/index.html

Structurally these two top-level scaffolds have not much in common, except being multi-cyclic, but as has been outlined previously compounds containing these structures have been identified to have effect on cancer cells. Even though they may induce different MOAs, their overall phenotypic endpoint when exposed onto cancer cell lines may be similar, inducing a cytostatic or cytotoxic effect.

Possible Compounds with Similar Mechanism of Actions

This analysis can be the starting point of *de novo* drug design studies. To showcase this, we performed a small virtual screening across PubChem for the two identified higher level scaffolds. These identified compounds likely have a similar MOA on the same biological system as the here investigated 38 drugs, but likely still act differently across different biological systems. The top 3 identified compounds for each scaffold are listed in supplementary table 3.

The identified compounds for each of the two scaffolds are closely related to the identified set of 38 drugs. As expected, the three drugs strongly associated to the first scaffold (phenothiazine, trimeprazine, and perphenazine) have antipsychotics & antiemetic functions, while the ones associated with the second scaffold (oleandrin, convallatoxin, and deslanoside) are antiarrhythmic agent. This supports our previous statements, linking these substructures to the listed functional groups.

Scaffold ID	Scaffold SMILE	Top 3 Hits on PubChem	Compound Category
1 (A)	c1ccc2c(c1)Nc1cccc c1S2	Phenothiazine	antipsychotic
		Trimeprazine	antiemetic
		Perphenazine	antiemetic, antipsychotic
2 (B)	O=C1C=C(C2CCC3 C2CCC2C4CCC(O5 CCCCO5)CC4CCC 23)CO1	Oleandrin	antiarrhythmic agent
		Convallatoxin	antiarrhythmic agent
		Deslanoside	antiarrhythmic agent

Table 3: For each of the 2 top-level scaffolds the 3 top PubChem compounds containing this substructure were retrieved, excluding compounds already included in the set of 676 compounds.

Description of the Identified Nanomaterials

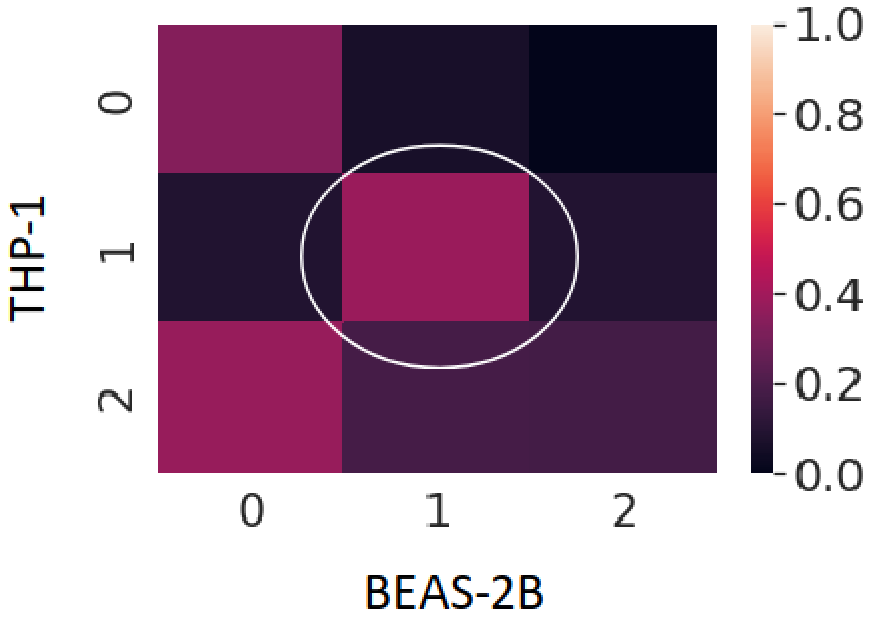

Figure 28: Jaccard Index between the THP-1 and BEAS-2B clusters. THP-1 cluster 1 and BEAS-2B cluster 1 show the highest Jaccard Similarity of 0.385.

Au20 COOH
Au20 PEG
Au5 PEG
QD COOH
QD PEG

Table 4: The overlapping nanomaterials between THP-1 cluster 1 and BEAS-2B cluster 1.

THP-1 cluster 1 and BEAS-2B cluster 1 share the highest Jaccard Similarity. Nanomaterials in the cluster comprise different functionalisations and dimensions of gold nanoparticles and quantum dots. Gold (Au) and quantum dots (QD) both have unique optical and electronic properties, for which they have often been preferred to be applied for various applications, either singularly or in combination (Ellis *et al.*, 2020; Liu *et al.*, 2006). They are both semiconductor nanocrystals therefore largely used for diagnostics and cell marking. Nanogold toxicity has long been studied, and discordant evidence is still present to date (Sukhanova *et al.*, 2022). However, it is now known that nanogold is internalized by endocytosis, a generic mechanism of cell uptake which is common to many other nanoparticles of the same shape (Alkilany and Murphy, 2010). Quantum dots are usually regarded as toxic nanoparticles, although it is currently known that this is modulated by the size, surface and core chemistry. Indeed, in previous analysis of the data used in this investigation, all the materials in this cluster were considered to have a high toxic profile

(integrating immunotoxicity, cytotoxicity and genotoxicity), with the exception of Au20 nanoparticles that were classified as medium hazard (Fortino *et al.*, 2022). It is reasonable to think that in case of highly toxic materials, the stress response of a cell system may be less heterogeneous, possibly contributing to the similarity observed between THP1 and BEAS-2B.

Identifying the Most Similar Chemical for each Nanomaterial Based on their Mechanism of Action

**Nanomaterial**	**Chemical**
Ag_COOH (gold)	ciclopirox
Ag_NH (silver)	lasalocid
Ag_PEG (silver)	ciclopirox
Au20_COOH (gold)	X15.delta.prostaglandin.J2
Au20_NH (gold)	pyrvinium
Au20_PEG (gold)	nadide
Au5_COOH (gold)	imipramine
Au5_NH (gold)	X15.delta.prostaglandin.J2
Au5_PEG (gold)	oxyphenbutazone
CuO_COOH (copper oxide)	furazolidone
CuO_core (copper oxide)	lycorine
CuO_NH (copper oxide)	lycorine
CuO_PEG (copper oxide)	flumetasone
MWCNT_COOH (multi-walled carbon nanotube)	X15.delta.prostaglandin.J2
MWCNT_core (multi-walled carbon nanotube)	triamterene
MWCNT_NH (multi-walled carbon nanotube)	thioridazine
MWCNT_PEG (multi-walled carbon nanotube)	ciclopirox

ND_COOH (nanodiamond)	flunisolide
ND_NH (nanodiamond)	phenazopyridine
ND_PEG (nanodiamond)	atropine.methonitrate
QD_COOH (quantum dot)	dilazep
QD_NH (quantum dot)	meglumine
QD_PEG (quantum dot)	beta.escin
TiO2r_COOH (titanium dioxide)	fenbendazole
TiO2r_core (titanium dioxide)	puromycin
TiO2r_NH (titanium dioxide)	ciclopirox
TiO2r_PEG (titanium dioxide)	primaquine
TiO2s_COOH (titanium dioxide)	nortriptyline
TiO2s_core (titanium dioxide)	puromycin
TiO2s_NH (titanium dioxide)	X15.delta.prostaglandin.J2
TiO2s_PEG (titanium dioxide)	helveticoside

Table 5: For each Fortino et al. the CMAP compound with the most similar transcriptomic alteration profile based on the KNeMAP vector, across all biological systems is displayed.

Small molecules and nanomaterials have important differences, including their physicochemical properties and the way in which they interact with biological entities. Indeed, nanomaterials are usually internalized and in a very unspecific way and induce generic cell stress responses, whereas drugs and other bulk compounds act on specific targets and usually are internalized by receptor recognition. Regardless of these differences, it has already been proved in other settings that the cross-talk between disciplines facilitates advancements and improves the predictions of the effect of nanomaterials on health (Kinaret *et al.*, 2020). The fast-pacing production and commercialisation of nanomaterials highlights the need for streamlining their safety assessment. In this light, highlighting commonalities with other mechanisms of action of known compounds, can provide important insights for their evaluation. Here, we compared a panel of 31 nanomaterials with CMAP compounds, and highlighted similarities based on their induced transcriptomic alterations. Five examples are reported hereafter.

CuO NH_2

Copper oxide (CuO) is a highly versatile nanomaterial with widespread applications in commercially available products. For this reason, multiple studies have described its potential effects on biological systems. Lycorine, instead, is a natural alkaloid used in clinics as an acetylcholinesterase (AChE) inhibitor (Kola *et al.*, 2023). AChE plays an important role

in the cholinergic system including nerve impulse transmission in synapses (Modesto and Martinez, 2010). Copper oxide is known for its potential effect on nervous systems, and levels of AChE have been found significantly decreased in various tissues upon its exposure (Sezer Tuncsoy *et al.*, 2019; Ganesan *et al.*, 2016). Importantly, both agents have been hypothesized to have antimicrobial activities (Bendaif *et al.*, 2018; Bezza *et al.*, 2020). The functional characterization of CuO NH2 reported in the original nanosolution study, confirms that this nanomaterial can affect various phases of the cell cycles and interfere with membrane homeostasis (Kinaret *et al.*, 2021).

Ag

Ciclopirox is a medication that has wide-ranging effects against fungal infections, as well as antibacterial and anti-inflammatory properties. Its method of action is believed to involve the chelation of polyvalent metal cations, which can inhibit numerous enzymes, including cytochromes. This, in turn, disrupts cellular processes such as mitochondrial electron transport and energy production (Sonthalia *et al.*, 2019). Silver (Ag) nanoparticles have been utilized with various medical applications, including antimicrobial and anticancer treatments. These nanoparticles operate by releasing silver ions (Ag^+) and generating reactive oxygen species, which cause changes to cytochrome and mitochondrial functions and ultimately lead to destruction of the membrane structure (Xu *et al.*, 2020).

Au5 PEG

Oxyphenbutazone (National Center for Biotechnology Information) was previously commercialized as a non-steroidal anti-inflammatory drug and analgesic to alleviate symptoms such as pain, swelling and stiffness associated with arthritis and gout. However, due to its association with blood dyscrasias and Stevens-Johnson syndrome, it was withdrawn from the market in 1984. Gold nanoparticles are frequently employed for their unique properties, and more recently, surface modifications have been utilized to enhance material performance and compatibility with biological systems. PEGylation, for example, can prevent the formation of a protein corona, thereby reducing recognition by the immune system. Gold compounds have been employed as therapeutic agents for the treatment of inflammatory diseases such as rheumatoid arthritis, and pegylated gold nanoparticles exhibit even greater potential for anti-inflammatory and anti-angiogenic activities (Hornos Carneiro and Barbosa, 2016).

MWCNT

Thioridazine is an antipsychotic medication that is no longer available for commercial use due to its adverse effects. Thioridazine has been associated with rare cases of acute cholestatic liver injury. Moreover, liver test abnormalities have been reported in cases of long-term use, although the exact biological mechanism remains unclear (Thioridazine, 2012). Similarly, multi-walled carbon nanotubes (MWCNTs) have long been shown to cause hepatotoxicity, including nonalcoholic steatohepatitis, fibrosis, liver cancer, and metabolic disorders, with mechanisms that are widely studies in the nanosafety field (Sun *et al.*, 2021; Ji *et al.*, 2009).

TiO2

Nortriptyline is a tricyclic antidepressant drug that is used for the short-term treatment of various types of depression. The mechanism of action is based on blocking the norepinephrine presynaptic receptors, which prevents the reuptake of this neurotransmitter

and increases its concentration in the CNS synaptic cleft (Merwar *et al.*, 2023). Titanium dioxide nanoparticles (TiO2) are the most commonly produced nanomaterials due to their industrial usefulness (Kinaret *et al.*, 2021). Naima et al. found that TiO2 nanoparticles administration in rats resulted in significant disruptions of their cognitive capacity and emotional reactivity, compared to the control groups. These behavioral effects were linked to changes in brain neurotransmitter levels, as well as enzyme alterations caused by the oxidative stress induced in the brain (Naima *et al.*, 2021).

Bibliography

Alanis-Lobato,G. *et al.* (2017) HIPPIE v2.0: enhancing meaningfulness and reliability of protein-protein interaction networks. *Nucleic Acids Res.*, **45**, D408–D414.

Alkilany,A.M. and Murphy,C.J. (2010) Toxicity and cellular uptake of gold nanoparticles: what we have learned so far? *J. Nanopart. Res.*, **12**, 2313–2333.

Aynaud,T. (2020) python-louvain 0.13: Louvain algorithm for community detection. GitHub.

Bendaif,H. *et al.* (2018) Antibacterial activity and virtual screening by molecular docking of lycorine from Pancratium foetidum Pom (Moroccan endemic Amaryllidaceae). *Microb. Pathog.*, **115**, 138–145.

Benjamini,Y. and Hochberg,Y. (1995) Controlling the false discovery rate: a practical and powerful approach to multiple testing. *Journal of the Royal Statistical Society: Series B (Methodological)*, **57**, 289–300.

Bezza,F.A. *et al.* (2020) Fabrication of monodispersed copper oxide nanoparticles with potential application as antimicrobial agents. *Sci. Rep.*, **10**, 16680.

Davis,A.P. *et al.* (2021) Comparative Toxicogenomics Database (CTD): update 2021. *Nucleic Acids Res.*, **49**, D1138–D1143.

Ellis,G.A. *et al.* (2020) Quantum dots and gold nanoparticles as scaffolds for enzymatic enhancement: recent advances and the influence of nanoparticle size. *Catalysts*, **10**, 83.

Federico,A. *et al.* (2022) Integrated Network Pharmacology Approach for Drug Combination Discovery: A Multi-Cancer Case Study. *Cancers (Basel)*, **14**.

Fortino,V. *et al.* (2022) Biomarkers of nanomaterials hazard from multi-layer data. *Nat. Commun.*, **13**, 3798.

Gallud,A. *et al.* (2020) Multiparametric profiling of engineered nanomaterials: unmasking the surface coating effect. *Adv Sci (Weinh)*, **7**, 2002221.

Ganesan,S. *et al.* (2016) Acute and sub-lethal exposure to copper oxide nanoparticles causes oxidative stress and teratogenicity in zebrafish embryos. *J. Appl. Toxicol.*, **36**, 554–567.

Gautier,L. *et al.* (2004) affy--analysis of Affymetrix GeneChip data at the probe level. *Bioinformatics*, **20**, 307–315.

Hornos Carneiro,M.F. and Barbosa,F. (2016) Gold nanoparticles: A critical review of therapeutic applications and toxicological aspects. *J. Toxicol. Environ. Health B Crit. Rev.*, **19**, 129–148.

Howe,K.L. *et al.* (2021) Ensembl 2021. *Nucleic Acids Res.*, **49**, D884–D891.

Jassal,B. *et al.* (2020) The Reactome Pathway Knowledgebase. *Nucleic Acids Res.*, **48**, D498–D503.

Ji,Z. *et al.* (2009) The hepatotoxicity of multi-walled carbon nanotubes in mice. *Nanotechnology*, **20**, 445101.

Kanehisa,M. and Goto,S. (2000) KEGG: Kyoto encyclopedia of genes and genomes. *Nucleic Acids Res.*, **28**, 27–30.

Kanehisa,M. *et al.* (2017) KEGG: new perspectives on genomes, pathways, diseases and drugs. *Nucleic Acids Res.*, **45**, D353–D361.

Kim,S. *et al.* (2019) PubChem 2019 update: improved access to chemical data. *Nucleic Acids Res.*, **47**, D1102–D1109.

Kinaret,P.A.S. *et al.* (2020) Covid-19 acute responses and possible long term consequences: What nanotoxicology can teach us. *Nano Today*, **35**, 100945.

Kinaret,P.A.S. *et al.* (2021) Toxicogenomic profiling of 28 nanomaterials in mouse airways. *Adv Sci (Weinh)*, **8**, 2004588.

Kola,A. *et al.* (2023) A Comparative Study between Lycorine and Galantamine Abilities to Interact with AMYLOID β and Reduce In Vitro Neurotoxicity. *Int. J. Mol. Sci.*, **24**.

Lamb,J. *et al.* (2006) The Connectivity Map: using gene-expression signatures to connect small molecules, genes, and disease. *Science*, **313**, 1929–1935.

Liu,N. *et al.* (2006) Hybrid gold/silica/nanocrystal-quantum-dot superstructures: synthesis and analysis of semiconductor-metal interactions. *J. Am. Chem. Soc.*, **128**, 15362–15363.

López,Y. *et al.* (2015) HitPredict version 4: comprehensive reliability scoring of physical protein-protein interactions from more than 100 species. *Database (Oxford)*, **2015**.

Marbach,D. *et al.* (2012) Wisdom of crowds for robust gene network inference. *Nat. Methods*, **9**, 796–804.

Martens,M. *et al.* (2021) WikiPathways: connecting communities. *Nucleic Acids Res.*, **49**, D613–D621.

Marwah,V.S. *et al.* (2019) eUTOPIA: solUTion for Omics data PreprocessIng and Analysis. *Source Code Biol. Med.*, **14**, 1.

Merwar,G. *et al.* (2023) Nortriptyline. In, *StatPearls*. StatPearls Publishing, Treasure Island (FL).

Mi,H. *et al.* (2021) PANTHER version 16: a revised family classification, tree-based classification tool, enhancer regions and extensive API. *Nucleic Acids Res.*, **49**, D394–D403.

Modesto,K.A. and Martinez,C.B.R. (2010) Roundup causes oxidative stress in liver and inhibits acetylcholinesterase in muscle and brain of the fish Prochilodus lineatus. *Chemosphere*, **78**, 294–299.

Naima,R. *et al.* (2021) Acute titanium dioxide nanoparticles exposure impaired spatial cognitive performance through neurotoxic and oxidative mechanisms in Wistar rats. *Biomarkers*, **26**, 760–769.

National Center for Biotechnology Information PubChem Compound Summary for CID 4641, Oxyphenbutazone. . *National Center for Biotechnology Information*.

Pavel,A *et al.* (2021) Integrated network analysis reveals new genes suggesting COVID-19 chronic effects and treatment. *Brief. Bioinformatics*.

Pavel,A., Serra,A., *et al.* (2022) Network analysis of microarray data. *Methods Mol. Biol.*, **2401**, 161–186.

Pavel,A., Saarimäki,L.A., *et al.* (2022) The potential of a data centred approach & knowledge graph data representation in chemical safety and drug design. *Comput. Struct. Biotechnol. J.*, **20**, 4837–4849.

Pavel,Alisa *et al.* (2021) Volta: adVanced mOLecular neTwork Analysis. *Bioinformatics*, **37**, 4587–4588.

Saarimäki,L.A. *et al.* (2021) Manually curated transcriptomics data collection for

toxicogenomic assessment of engineered nanomaterials. *Sci. Data*, **8**, 49.
Sayers,E.W. *et al.* (2022) Database resources of the national center for biotechnology information. *Nucleic Acids Res.*, **50**, D20–D26.
Scott,O.B. and Edith Chan,A.W. (2020) ScaffoldGraph: an open-source library for the generation and analysis of molecular scaffold networks and scaffold trees. *Bioinformatics*, **36**, 3930–3931.
Seabold,S. and Perktold,J. (2010) Statsmodels: Econometric and Statistical Modeling with Python. In, *Proceedings of the 9th Python in Science Conference*, Proceedings of the python in science conference. SciPy, pp. 92–96.
Sezer Tuncsoy,B. *et al.* (2019) Effects of Copper Oxide Nanoparticles on Tissue Accumulation and Antioxidant Enzymes of Galleria mellonella L. *Bull. Environ. Contam. Toxicol.*, **102**, 341–346.
Sonthalia,S. *et al.* (2019) Topical ciclopirox olamine 1%: revisiting a unique antifungal. *Indian Dermatol. Online J.*, **10**, 481–485.
Sukhanova,A. *et al.* (2022) Dependence of quantum dot toxicity in vitro on their size, chemical composition, and surface charge. *Nanomaterials (Basel)*, **12**.
Sun,T. *et al.* (2021) Nanomaterials and hepatic disease: toxicokinetics, disease types, intrinsic mechanisms, liver susceptibility, and influencing factors. *J. Nanobiotechnology*, **19**, 108.
The Gene Ontology Consortium (2021) The Gene Ontology resource: enriching a GOld mine. *Nucleic Acids Res.*, **49**, D325–D334.
Thioridazine (2012) In, *LiverTox: Clinical and Research Information on Drug-Induced Liver Injury*. National Institute of Diabetes and Digestive and Kidney Diseases, Bethesda (MD).
Thomas,P.D. *et al.* (2003) PANTHER: a library of protein families and subfamilies indexed by function. *Genome Res.*, **13**, 2129–2141.
Virtanen,P. *et al.* (2020a) Author Correction: SciPy 1.0: fundamental algorithms for scientific computing in Python. *Nat. Methods*, **17**, 352.
Virtanen,P. *et al.* (2020b) SciPy 1.0: fundamental algorithms for scientific computing in Python. *Nat. Methods*, **17**, 261–272.
Waskom,M. *et al.* (2018) mwaskom/seaborn: v0.9.0 (July 2018). *Zenodo*.
Wishart,D.S. *et al.* (2008) DrugBank: a knowledgebase for drugs, drug actions and drug targets. *Nucleic Acids Res.*, **36**, D901-6.
Wishart,D.S. *et al.* (2018) DrugBank 5.0: a major update to the DrugBank database for 2018. *Nucleic Acids Res.*, **46**, D1074–D1082.
Xu,L. *et al.* (2020) Silver nanoparticles: Synthesis, medical applications and biosafety. *Theranostics*, **10**, 8996–9031.
